# Neurotransmitter Dysregulation in Parkinson’s Disease: Pathophysiological Insights and Therapeutic Perspectives

**DOI:** 10.1021/acschemneuro.5c00809

**Published:** 2026-03-29

**Authors:** Feyza Sule Aslan, Beyza Barut, Sudenaz Ozturk, Tugba Eyigurbuz, Enes Akyuz

**Affiliations:** † School of Medicine, 64050 Marmara University, Istanbul 34854, Türkiye; ‡ School of Medicine, 162334 Tekirdağ Namık Kemal University, Tekirdağ 59030, Türkiye; § Department of Neurology, 162307 Bağcılar Training and Research Hospital, Istanbul 34200, Türkiye; ∥ Department of Pediatrics, School of Medicine, University of Wisconsin-Madison, Madison, Wisconsin 53706, United States; ⊥ Department of Biophysics, International School of Medicine, 448249 University of Health Sciences, Istanbul 34668, Türkiye

**Keywords:** receptors, neuroimaging, neurotransmission, α-synuclein

## Abstract

Parkinson’s disease (PD) is a prevalent neurodegenerative movement disorder characterized by bradykinesia, rigidity, and resting tremor, progressing insidiously over time. Central to its pathophysiology is the degeneration of dopaminergic neurons in the substantia nigra, leading to a significant decrease in striatal dopamine (DA) levels. This dopaminergic deficit disrupts basal ganglia circuitry, impairing motor function and contributing to the core symptoms of the disease. While the etiology of PD remains incompletely understood, a combination of genetic predispositions and environmental exposures has been implicated. Beyond dopaminergic dysfunction, emerging evidence suggests that other neurotransmitter systems, including noradrenergic, serotonergic, cholinergic, glutamatergic, and γ-aminobutyric acidergic (GABAergic) pathways, are also involved in disease progression and symptom heterogeneity. Pathological hallmarks such as α-synuclein (α-syn) misfolding and Lewy body (LB) formation, along with mitochondrial dysfunction, oxidative stress, and neuroinflammation, further exacerbate neurodegeneration and neurotransmitter imbalances. Despite advances in symptomatic treatment, current therapies primarily target DA deficiency and fail to reverse neurodegenerative processes. The involvement of multiple neurotransmitter systems highlights the complex neurochemical landscape of PD and underscores the need for multifaceted therapeutic strategies. Understanding the broader role of neurotransmitters in PD pathogenesis offers promising avenues for disease-modifying interventions and improved symptom management. This review summarizes the recent findings on the contribution of various neurotransmitters to PD, emphasizing their potential as targets for future therapeutic development. By integrating the current literature, we aim to provide a comprehensive overview of neurotransmitter involvement in PD and its implications for advancing treatment paradigms.

## Graphical abstract

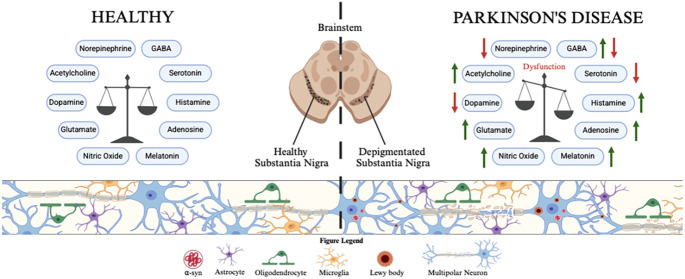

## 1 Introduction

PD is the most common neurodegenerative movement disorder characterized by bradykinesia, rigidity, and resting tremor, with a slowly progressive clinical course.^1,2^ Although the exact cause of the disease remains unknown, genetic mutations and environmental factors are believed to contribute to its pathogenesis.^3^ PD is primarily characterized by striatal DA deficiency resulting from the progressive degeneration of dopaminergic neurons in the substantia nigra, a key component of the basal ganglia involved in the modulation and coordination of movement.^1,4^ One of the hallmark pathological features of the disease is the misfolding of the α-syn protein, which aggregates to form LBs.^5,6^ Additionally, mitochondrial dysfunction, neuroinflammation, oxidative stress, and disruptions in neurotransmitter systems are also implicated in PD pathogenesis.^7,8^

Neurotransmitters, which mediate communication between neurons in the brain, are released into the synaptic cleft and modulate presynaptic and postsynaptic cell activity in a highly regulated manner, depending on the type of neurotransmitter. Through synaptic transmission, neurotransmitters contribute to neuronal excitation or inhibition.^9^ DA, a critical neurotransmitter for optimal behavioral and cognitive performance, provides motor modulation. Alterations in DA levels lead to imbalances within the basal ganglia, resulting in motor dysfunction.^10^ Disruption of dopaminergic system homeostasis is a key contributor to the development of neurodegenerative disorders such as PD.^8^

Although DA deficiency represents the core pathophysiological feature of PD, studies have shown that neurodegeneration also affects noradrenergic, serotonergic, and cholinergic neuronal populations.^11^ Furthermore, imbalances in other neurotransmitter systems—such as excessive glutamatergic activity and reduced GABA-mediated inhibition—contribute to the diversity and severity of symptoms observed in PD.^12^ While current therapeutic approaches can effectively manage motor symptoms, they have limited impact on disease progression.^13^ Therefore, understanding the role of neurotransmitters in PD is essential for developing novel therapeutic strategies aimed at altering the disease course. This review aims to present the contribution of neurotransmitters to PD pathogenesis and explore their potential roles in future treatment options based on the latest literature.

## 2 Glutamate

Glutamate is the primary excitatory neurotransmitter in the central nervous system (CNS), contributing to synaptic transmission and plasticity.^14^ This excitatory neurotransmitter exerts its effects primarily through ionotropic (iGluRs) and metabotropic (mGluRs) receptors and is crucial for modulating behavior, perception, and cognition.^15,16^ Both iGluRs and mGluRs are further classified based on their pharmacological and electrophysiological properties.^17^ iGluRs include α-amino-3-hydroxy-5-methyl-4-isoxazolepropionic acid receptors (AMPARs), kainate receptors (KARs), and *N*-methyl-d-aspartate receptors (NMDARs), whereas the G-protein-coupled mGluRs are divided into subtypes mGluR1 through mGluR8.^18^ mGluRs, which are highly expressed in the basal ganglia, regulate neuronal excitability, neurotransmitter release, and synaptic plasticity.^19^

To prevent excitotoxicity, defined as the neuronal and glial death resulting from excessive or prolonged glutamate receptor activation, glutamate homeostasis is tightly regulated. After fulfilling its synaptic function, glutamate is cleared from the synaptic cleft by high-affinity excitatory amino acid transporters (EAATs) located on neurons and glial cells.^20,21^ Five EAAT subtypes have been identified: EAAT1, EAAT2, EAAT3, EAAT4, and EAAT5. EAAT1 is predominantly expressed in astrocytes, while EAAT2 (also known as glutamate transporter-1 (GLT-1)) is found in both astrocytes and neurons.^22,23^ Dysregulation of the glutamate cycle leading to glutamate hyperactivity and excitotoxicity has been implicated in the pathogenesis of neurodegenerative disorders such as PD.^24^

L-3,4-dihydroxyphenylalanine (L-DOPA), a DA precursor, is the mainstay treatment for PD, though it frequently induces dyskinesias: abnormal, involuntary movements. In a 6-hydroxydopamine (6-OHDA)-induced PD mouse model, suppression of presynaptic corticostriatal glutamatergic activity was shown to alleviate L-DOPA-induced dyskinesia (LID), underscoring the critical role of glutamatergic neurons in LID pathophysiology.^14^ Reducing synaptic glutamate by either inhibiting glutamatergic neuronal activity or upregulating GLT-1 expression has been proposed as a potential therapeutic approach to manage LID.^25^ Furthermore, safinamide, a glutamate modulator, has demonstrated the ability to reduce the glutamatergic synaptic transmission in the striatal network. In experimental PD models, safinamide optimized the effects of L-DOPA by reducing the excitability of striatal spiny projection neurons and modulating the synaptic transmission. The combined use of safinamide and L-DOPA may yield favorable outcomes in managing PD-related motor disturbances.^26^

Glycation, a post-translational modification that alters protein structure and function with aging, has also been implicated in PD. An animal study involving the glycating agent methylglyoxal investigated its effect on glutamatergic transmission and α-syn aggregation. Glycation was shown to accelerate PD-like cognitive, sensory, and motor impairments, possibly via enhanced glutamatergic signaling. These findings suggest that antiglycation and antiglutamatergic agents may hold promise as disease-modifying therapies for PD.^27^

Among the key contributors to synaptic dysfunction in PD is the dysregulated activation of NMDARs. Abnormal NMDAR activity has been recognized as a significant factor in PD pathogenesis, and NMDAR antagonists are being explored as potential therapeutic agents.^28^ In a 6-OHDA-induced PD rat model, the effects of memantine, a well-known NMDAR antagonist, on LID were evaluated. While the blockade of NMDARs showed only temporary efficacy, the results were consistent with clinical observations.^29^ Another animal study examined the effects of ketamine, an NMDAR antagonist, on short-term memory deficits and depressive-like behaviors in a PD animal model. In rats with bilateral lesions of the substantia nigra pars compacta (SNpc), ketamine reversed both cognitive and mood-related symptoms, suggesting its potential utility in treating nonmotor symptoms of PD.^30^

Beyond NMDARs, dysregulation of other glutamate receptors such as mGluRs has also been implicated in PD.^31^ Metabotropic glutamate receptor 3 (mGluR3), in particular, has emerged as a potential therapeutic target due to its role in modulating synaptic function and reducing neuroinflammation. Both *in vivo* and clinical studies have shown that mGluR3 activation exerts neuroprotective effects and promotes cortical plasticity in PD models. Genetic variations in GRM3, the gene encoding mGluR3, may also contribute to PD susceptibility. Selective mGluR3 ligands may thus serve as promising disease-modifying agents in PD therapy.^32^

Glutamate transporters, like glutamate receptors, also play a critical role in PD research.^33^ Wnt1, a protein involved in neural development, has been shown to promote EAAT2 expression and support astrocyte-mediated neuroprotection in PD. The transcription factor nuclear factor-κB (NF-κB), known for its involvement in synaptic plasticity, may regulate EAAT2 expression under the influence of Wnt1.^34,35^ In a 1-methyl-4-phenyl-1,2,3,6-tetrahydropyridine (MPTP)-induced PD animal model, ceftriaxone, a β-lactam antibiotic known to enhance GLT-1 expression, exhibited neuroprotective and behavioral benefits, including improved cognitive function. These findings support the therapeutic potential of ceftriaxone in preventing or treating PD-related dementia.^36^

Mutations in *PINK1*, a gene crucial for neuronal homeostasis and mitochondrial function, have also been linked to synaptic dysfunction in PD.^37^ In familial early onset PD (EOPD), loss-of-function mutations in *PINK1* have been associated with increased levels of glutamatergic transmission in dorsal striatal spiny projection neurons. *In vivo* studies indicate that *Pink1* is essential for maintaining normal glutamatergic signaling within striatal circuits, implicating it in the mechanisms underlying striatal dysfunction in PD.^38^

Rapamycin, a mechanistic target of the rapamycin (mTOR) inhibitor, has been shown to preserve glutamate uptake and transporter expression in astrocytes. These effects may be mediated through glial and anti-inflammatory mechanisms, contributing to the neuroprotective properties of rapamycin (Figure 1A).^39^

**Figure 1:**
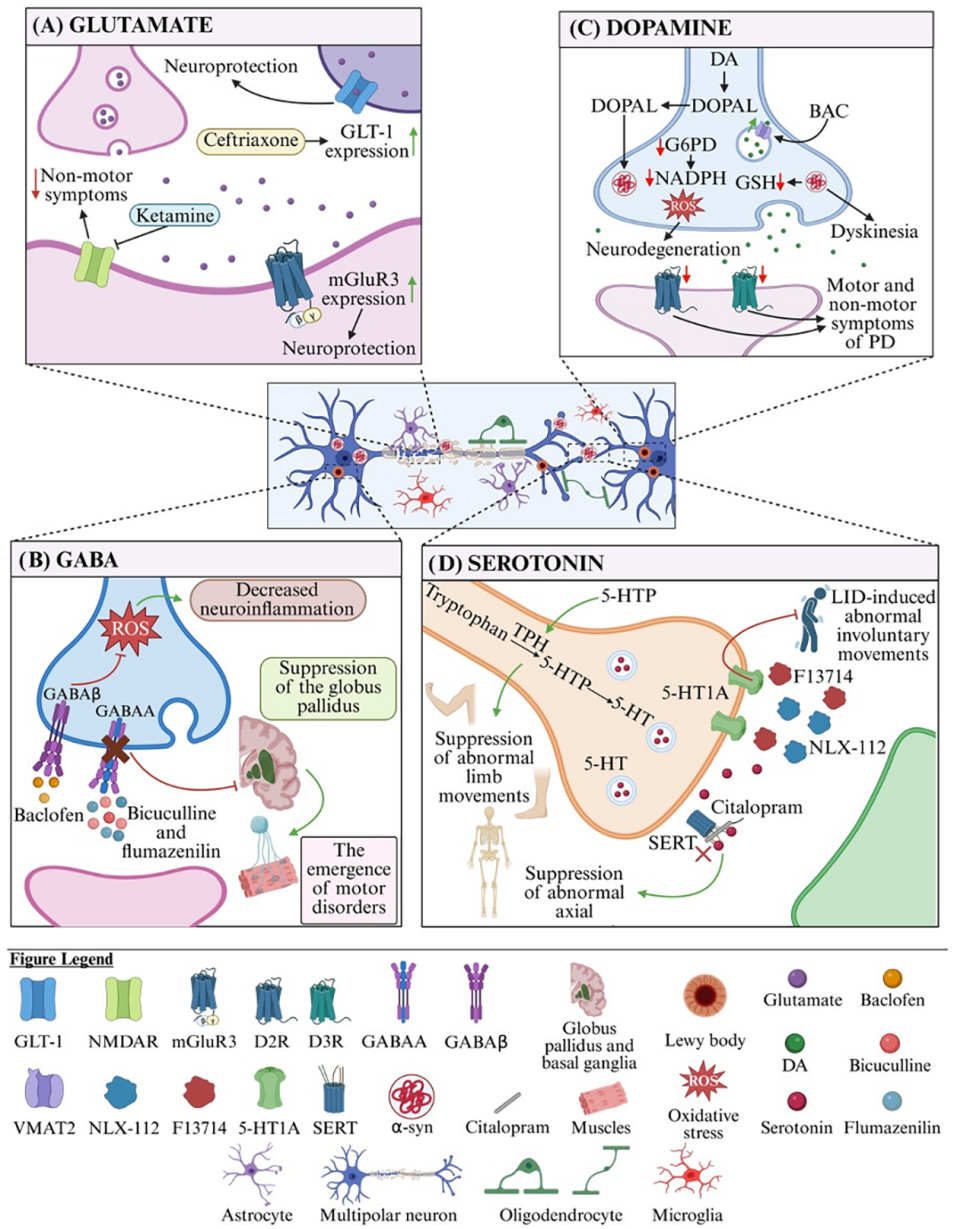
(A) The role of glutamate in PD. Ceftriaxone by increasing the expression of GLT-1, shows neuroprotective and behavioral benefits, including improved cognitive function.^36^ An NMDAR antagonist ketamine reversed both cognitive and mood-related symptoms in rats with bilateral lesions of the SNpc suggesting its potential utility in treating nonmotor symptoms of PD.^30^ mGluR3 activation in neurons shows neuroprotective effects and promotes cortical plasticity in PD models. Genetic variations in GRM3, the gene encoding mGluR3, may also contribute to PD susceptibility suggesting that selective mGluR3 ligands may thus serve as promising disease-modifying agents in PD therapy.^32^ (B) The role of GABA in PD. Blockade of GABA_A_ receptors in the globus pallidus of PD rats using antagonists such as bicuculline and flumazenil reversed typical motor deficits, indicating that modulation of GABAergic neurotransmission holds therapeutic relevance in treating PD-related motor impairment.^54^ The GABA_B_ receptor agonist baclofen prevented MPTP-induced toxicity via GABAergic pathways by inhibiting neuroinflammation and oxidative stress, highlighting its anti-inflammatory activity as a potential neuroprotective strategy in PD.^53^ (C) The role of DA in PD. VMAT2 upregulation via BAC improves DA release and attenuates neurodegeneration in PD. The observed increase in DA release and neuronal protection in VMAT2-overexpressing mice suggests that interventions aimed at enhancing vesicular capacity may offer therapeutic benefits in PD.^75^ Elevated levels of DOPAL (a highly reactive DA metabolite) have been linked to disrupted proteostasis and degeneration of neuronal projections via α-syn-related mechanisms in PD. DOPAL-induced α-syn accumulation impairs neuronal homeostasis, leading to dopaminergic neuron loss and motor dysfunction.^73^ G6PD maintains redox balance through NADPH production;^77^ its deficiency can increase oxidative stress, leading to neurodegeneration. α-syn accumulation disrupts metabolic flux, reduces NADPH and GSH levels, promotes DA oxidation, and lowers DA concentration.^78^ A clinical study using PET imaging with the D_2_R/D_3_R ligand [18F] fallypride assessed striatal and extrastriatal D_2_R and D_3_R expression. It revealed that in the putamen and globus pallidus the severity of motor symptoms is positively correlated with D_2_R and D_3_R density.^71^ (D) The role of 5-HT in PD. The biased presynaptic 5-HT1A agonist F13714 demonstrated potent antidyskinetic activity and nearly completely suppressed abnormal involuntary movements associated with LID, highlighting the need to develop selective, full agonists of 5-HT receptors for treating PD patients suffering from peak-dose LID.^87^ NLX-112 prevented the development of LID by activating presynaptic autoreceptors in the raphe nuclei, suggesting that targeting 5-HT1A receptors in these specific brain regions may offer a promising therapeutic strategy for managing dyskinesia.^88^ 5-HTP and citalopram attenuated the development of LID by suppressing different subtypes of abnormal involuntary movements, where citalopram primarily reduced axial movements and 5-HTP targeted limb movements. These differential effects may relate to the drugs’ specific impacts on L-DOPA-derived dopamine and 5-HT metabolism.^92^ *5-HT: serotonin, 5-HTP: 5-hydroxytryptophan, BAC: bacterial artificial chromosome*, *D*_2_ *R: D*_2_ *receptor*, *D*_3_ *R: D*_3_ *receptor*, *DA: dopamine*, *DOPAL: 3,4-dihydroxyphenylacetaldehyde*, *G6PD: glucose-6-phosphate dehydrogenase*, *GABA: γ-aminobutyric acid*, *GLT-1: glutamate transporter-1*, *GRM3: the mGluR3 receptor encoding gene, GSH: glutathione*, *mGluR3: metabotropic glutamate receptor 3*, *LID: L-DOPA induced dyskinesia, MPTP: 1-methyl-4-phenyl-1,2,3,6-tetrahydropyridine, NADPH: nicotinamide adenine dinucleotide phosphate*, *NMDAR: N-methyl-* d *-aspartate receptor*, *PD: Parkinson’s disease*, *SERT: serotonin transporter*, *SNpc: substantia nigra pars compacta,VAChT: vesicular acetylcholine transporter*, *VMAT2: vesicular monoamine transporter 2*, *α-syn: α- synuclein.*

Taken together, current evidence suggests that therapeutic targeting of glutamate receptors and transporters holds considerable promise for the treatment of PD. Moreover, elucidating the genetic mechanisms underlying PD pathophysiology may facilitate earlier diagnosis and the development of targeted therapies. A deeper understanding of glutamatergic involvement in PD is expected to significantly contribute to both diagnostic and therapeutic advancements (Table 1).

**Table 1:** Summary of the Findings Reporting the Role of Glutamate in PD^a^

S.N.	study type	PD model	therapeutic drug and dose	observation	remarks	references
1	*In vitro*	Astrocytes and neuroblastoma SH-SY5Y cells		Wnt1 has been shown to promote EAAT2 expression and support astrocyte-mediated neuroprotection in PD	NF-κB, known for its involvement in synaptic plasticity, may regulate EAAT2 expression under the influence of Wnt1	^35^
2	*In vitro*, *In vivo*	Primary cortical astrocytes of C57BL/6 mice, MPTP-induced mice, BV2 cells, PC12 cells	Rapamycin (100 nM)	Rapamycin has been shown to preserve glutamate uptake and transporter expression in astrocytes	These effects may be mediated through glial and anti-inflammatory mechanisms, contributing to the neuroprotective properties of rapamycin	^39^
3	*In vivo*	6-OHDA induced and L-DOPA (10 mg/kg, i.p.) plus Benserazide (12 mg/kg) treated C57BL/6Jnarl (wild-type) and vGluT2-Cre mice	Amantadine (60 mg/kg, i.p.), LDN 212320 (40 mg/kg, i.p.)	Suppression of presynaptic corticostriatal glutamatergic activity was shown to alleviate LID, underscoring the critical role of glutamatergic neurons in LID pathophysiology	Reducing synaptic glutamate by either inhibiting glutamatergic neuronal activity or upregulating GLT-1 expression has been proposed as a potential therapeutic approach to manage LID	^14^
4	*In vivo*	6-OHDA induced Wistar rats	l-DOPA plus Safinamide (1–100 μM)	Safinamide optimized the effects of L-DOPA by reducing the excitability of striatal spiny projection neurons and modulating synaptic transmission	The combined use of safinamide and L-DOPA may yield favorable outcomes in managing PD-related motor disturbances	^26^
5	*In vivo*	Transgenic Thy1-aSyn mice	MGO (31.6 mM)	Glycation was shown to accelerate PD-like cognitive, sensory, and motor impairments, possibly via enhanced glutamatergic signaling	These findings suggest that antiglycation and antiglutamatergic agents may hold promise as disease-modifying therapies for PD	^27^
6	*In vivo*	6-OHDA induced Sprague–Dawley rats	L-DOPA (6 mg/kg plus Benserazide 6 mg/kg, s.c.), Memantine (5–20 mg/kg, i.p.)	The effects of memantine on LID were evaluated	While the blockade of NMDARs showed only temporary efficacy, the results were consistent with clinical observations	^29^
7	*In vivo*	6-OHDA induced Wistar rats	Ketamine 5, 10, and 15 mg/kg (i.p., once a week) and Imipramine 20 mg/kg (i.p. daily)	Ketamine effects on short-term memory deficits and depressive-like behaviors were studied	In rats with bilateral lesions of the SNpc, ketamine reversed both cognitive and mood-related symptoms, suggesting its potential utility in treating nonmotor symptoms of PD	^30^
8	*In vivo*	MPTP-induced Wistar rats	Ceftriaxone (100 or 200 mg/kg/day)	Ceftriaxone exhibited neuroprotective and behavioral benefits, including improved cognitive function	These findings support the therapeutic potential of ceftriaxone in preventing or treating PD-related dementia	^36^
9	*In vivo*	*Pink1* knockout rats		EOPD, loss-of-function mutations in *PINK1* have been associated with increased glutamatergic transmission in dorsal striatal spiny projection neurons	*Pink1* is essential for maintaining normal glutamatergic signaling within striatal circuits, implicating it in the mechanisms underlying striatal dysfunction in PD	^38^
10	*In vivo*, *Observational* (*Cross-sectional*) *Human study*	MPTP-induced C57/Bl6J mice		mGluR3 activation exerts neuroprotective effects and promotes cortical plasticity in PD models, genetic variations in GRM3 may also contribute to PD susceptibility	Selective mGluR3 ligands may thus serve as promising disease-modifying agents in PD therapy	^32^
		PD patients (*n* = 723), HC (*n* = 826)				

a 6-OHDA: 6-hydroxydopamine, EAAT: excitatory amino acid transporter, EOPD: early onset Parkinson’s disease, GLT-1: glutamate transporter-1, GRM3: the mGluR3 receptor encoding gene, HC: healthy controls, L-DOPA: L-3,4-dihydroxyphenylalanine, LID: L-DOPA-induced dyskinesia, MPTP: 1-methyl-4-phenyl-1,2,3,6-tetrahydropyridine, mGluR: metabotropic glutamate receptor, NF-κB: nuclear factor-κB, NMDAR: *N*-methyl-d-aspartate receptor, PD: Parkinson’s disease, SNpc: substantia nigra pars compacta.

## 3 GABA

GABA is the main inhibitory neurotransmitter in the CNS, preventing excessive neuronal excitation by suppressing neuronal activity.^40^ The GABAergic system is composed of four key components: GABA itself, GABA transporters (GATs), GABAergic receptors, and GABAergic neurons.^41^ This system plays a pivotal role in the regulation of neurogenesis, neuronal maturation, and apoptosis, processes critical for proper CNS function, particularly in learning and memory.^41^ GABA is synthesized from glutamate by the enzyme glutamic acid decarboxylase (GAD) within GABAergic neurons.^40^ Astrocytes also contribute to maintaining the metabolic balance between GABA and glutamate. In astrocytes, glutamine is converted to glutamate by glutaminase, and subsequently to GABA by GAD. The concentration of GABA in the synaptic cleft is regulated through its interaction with GABA receptors.^42^

Synaptic inhibition is mediated and regulated by three types of GABA receptors: GABA_A_, GABA_B_, and GABA_C_.^43^ GABA_A_ is an ionotropic receptor that facilitates the influx of chloride ions (Cl^–^) into the cell upon GABA binding. This increase in intracellular Cl^–^ induces membrane hyperpolarization, producing a rapid inhibitory signal that effectively reduces neuronal excitability.^44^ In contrast, GABA_B_ is a metabotropic receptor that mediates slow and prolonged inhibitory effects through G-protein signaling, contributing to sustained changes in signal transduction and complex CNS processes.^45^ GABA_C_, structurally similar to GABA_A_ and often considered a subtype of it, is less widely expressed and is primarily involved in retinal inhibition.^46^

Inflammatory cytokines, oxidative stress, and amyloid β (Aβ) accumulation can impair synaptic GABAergic transmission.^41^ The resulting decrease in GABAergic activity can lead to excitatory/inhibitory imbalance in the CNS. Disruptions in GABAergic function are implicated in neurological disorders such as Alzheimer’s disease (AD), autism spectrum disorders, and PD.^47^ Changes in the GABAergic system in PD are of particular interest due to their neuroprotective and therapeutic implications.^48^ An observational human study investigating GABA levels in the upper brainstem revealed significantly reduced GABA and coregulated signals (GABA+) in PD patients compared to healthy controls (HC). Alterations in brainstem GABA+ levels may facilitate early detection of GABAergic dysfunction before the onset of nigrostriatal defects.^49^ Similarly, research examining EOPD found significant reductions in both cortical gyrification of the sensorimotor cortex and GABA concentrations, suggesting their potential as preclinical biomarkers.^50^ In another study on GABAergic changes in the thalamocortical circuitry of PD patients, GABA concentrations in the primary motor cortex were found to be inversely correlated with disease severity, regardless of dopaminergic medication status or motor symptom subtype (tremor-dominant or not).^51^ Furthermore, differences in GABA levels in the basal ganglia between postural instability gait difficulty (PIGD) and tremor-dominant (PDT+) PD subtypes have been demonstrated, indicating subtype-specific inhibitory motor dysfunctions.^52^

A rat study on GABA_B_ receptors has shown that in an MPTP-induced rat model of PD, the GABA_B_ receptor agonist baclofen inhibits neuroinflammation and oxidative stress, thereby preventing MPTP-induced toxicity via GABAergic pathways.^53^ In terms of GABA_A_ receptors, antagonists such as bicuculline and flumazenil have been found to reverse typical motor deficits when blocked in the globus pallidus of PD rats.^54^ Thus, modulation of GABAergic neurotransmission, particularly via GABA_A_ receptors, holds therapeutic relevance in PD.^54^

In a study utilizing *in vivo* ultrahigh-field 14.1 T ^1^H magnetic resonance spectroscopy (H-MRS) in 6-OHDA and α-syn-based PD rat models, GABA was investigated as a potential early biomarker. The 6-OHDA rats exhibited significant increases in GABA levels and decreases in glutamate and N-acetyl-aspartate (NAA) levels in the ipsilateral hemisphere compared with the contralateral side. In α-syn-overexpressing rats, only the striatal GABA levels were significantly increased. These findings suggest that striatal GABA level measurement via H-MRS could allow early detection of nigrostriatal deficits before neurodegeneration occurs.^55^ Another component of the GABAergic system, GABA transporters, also contributes to the PD pathophysiology. In a model of EOPD, striatal GABA transporter-1 (GAT-1) and GABA transporter-3 (GAT-3) were found to be downregulated in a dysregulated manner, leading to enhanced, prolonged GABA-mediated inhibition of DA release in the dorsal striatum. Targeting striatal GATs and astrocyte-mediated GABA-DA interactions may offer novel therapeutic strategies for modulating DA signaling in PD.^56^

Beyond the studies addressing GABA’s role in PD pathogenesis, research has also focused on its interactions with other neurotransmitter systems such as cholinergic and dopaminergic networks. Somatic symptom disorder (SSD), a core feature of the PD psychosis spectrum, has been associated with an elevated GABA content in the medial prefrontal cortex (mPFC). A study assessing basal GABA+ and glutamate levels in the mPFC across groups of SSD+PD, PD, HCs, and SSD individuals highlighted the role of GABAergic neurotransmission in SSD development within PD patients. These findings not only provide insight into the neurochemical substrates of SSD+PD but also contribute to the identification of new neuroprotective strategies.^57^

In PD, dual-transmitting cholinergic and GABAergic striatal interneurons (CGINs) exhibit a collapse of GABAergic inhibition. Under dopamine-depleted conditions, elevated intracellular Cl^–^ levels disrupt the balance within CGINs, resulting in the loss of GABAergic inhibition and an unopposed cholinergic excitatory influence. Therefore, agents targeting Cl^–^ cotransporters may be beneficial in alleviating PD symptoms (Figure 1B).^58^

In summary, the GABAergic system undergoes significant alterations in PD, contributing to excitatory/inhibitory imbalance and the progression of motor and cognitive dysfunction. Changes in GABA levels may serve as early biomarkers of the disease, while modulation of GABA receptors and transporters represents a promising therapeutic target (Table 2).

**Table 2:** Summary of the Findings Reporting the Role of GABA in PD^a^

s. n.	study type	PD model	therapeutic drug and dose	observation	remarks	references
1	*In vivo*	Swiss mice rodents (haloperidol)	Bicuculline (1 ve 5 mg/kg) ve Flumazenil (3 ve 6 mg/kg)	In terms of GABA_A_ receptors, antagonists such as bicuculline and flumazenil have been found to reverse typical motor deficits when blocked in the globus pallidus of PD rats.	Modulation of GABAergic neurotransmission, particularly via GABA_A_ receptors, holds therapeutic relevance in PD.	^54^
2	*In vivo*	6-OHDA and α-syn-based rat models		The 6-OHDA rats exhibited significant increases in GABA levels and decreases in glutamate and N-acetyl-aspartate levels in the ipsilateral hemisphere compared to the contralateral side. In α-syn-overexpressing rats, only striatal GABA levels were significantly increased.	Striatal GABA level measurement via H-MRS could allow early detection of nigrostriatal deficits before neurodegeneration occurs.	^55^
3	*In vivo*	C57BL/6 J mice		In a model of early onset parkinsonism, striatal GAT-1 and GAT-3 were found to be downregulated in a dysregulated manner, leading to enhanced, prolonged GABA-mediated inhibition of DA release in the dorsal striatum.	Targeting striatal GATs and astrocyte-mediated GABA-DA interactions may offer novel therapeutic strategies for modulating DA signaling in PD.	^56^
		*SNCA*+ and *Snca–/–* mice				
4	*In vivo*	Lhx6-iCre mice	Bumetanide (2–2.5 mg kg^–1^)	Under dopamine-depleted conditions, elevated intracellular Cl^–^ levels disrupt the balance within CGINs, resulting in the loss of GABAergic inhibition and an unopposed cholinergic excitatory influence.	Agents targeting Cl^–^ cotransporters may be beneficial in alleviating PD symptoms.	^58^
5	*In vivo*	MPTP rat	Baclofen (10 and 20 mg/kg) and GABA-B antagonist CGP35348 (10 mg/kg)	The GABA_B_ receptor agonist baclofen inhibits neuroinflammation and oxidative stress, thereby preventing MPTP-induced toxicity via GABAergic pathways	Baclofen is rich in anti-inflammatory activity via the GABAergic pathway.	^53^
6	*Observational Human Study*	PD patients (*n* = 18) HC (*n* = 18 age and sex-matched)		GABA levels in the upper brainstem revealed significantly reduced GABA and GABA in PD patients compared to healthy controls.	Alterations in brainstem GABA+ levels may facilitate early detection of GABAergic dysfunction before the onset of nigrostriatal defects.	^49^
7	*Human Study*	EOPD (*n* = 10), Late-onset Parkinson’s disease [LOPD]: 40		Significant reductions in cortical gyrification and GABA concentrations in the sensorimotor cortex have been detected in patients with PD.	It has been suggested that GABA and sensorimotor cortex MRI may be potential markers for the preclinical diagnosis of PD.	^50^
8	*Human Study*	PD patient (*n* = 60)		GABA concentrations in the primary motor cortex were found to be inversely correlated with disease severity, regardless of dopaminergic medication status or motor symptom subtype (tremor-dominant or not).	Cerebral GABA may have a protective effect at the neuronal level (e.g., by preventing calcium-based neurotoxicity) or at the circuit level (e.g., by preventing dysfunctional motor hyperactivity).	^51^
		HC (*n* = 22)				
9	*Human Study: Cohort*	PIGD (*n* = 13) and PDT+ (*n* = 9)		Differences in GABA levels in the basal ganglia between PIGD and PDT+ PD subtypes have been demonstrated, indicating subtype-specific inhibitory motor dysfunctions.	GABAergic dysfunction may have an important impact on the pathogenesis of PD.	^52^
		HC (*n* = 16)				
10	*Human Study: Cohort*	SSD+PD (*n* = 23)		A study assessing basal GABA+ and glutamate levels in the mPFC across groups of SSD+PD, PD, healthy controls (HC), and SSD individuals highlighted the effect of GABAergic neurotransmission in SSD development within PD patients.	The study not only provides insight into the neurochemical substrates of SSD+PD but also contributes to the identification of new neuroprotective strategies.	^57^
		Only PD (*n* = 19)				
		Only SSD (*n* = 14)				
		HC (*n* = 19)				

a CGINs: cholinergic and GABAergic striatal interneurons, Cl: Clorur, DA: dopamine, HC: healthy controls, H-MRS: ultrahigh-field 14.1 T ^1^H magnetic resonance spectroscopy, GABA: gama-aminobütirik asit, GABA+: GABA and coregulated signals, GAT: GABA transporter, MRI: magnetic resonance imaging, PD: Parkinson’s Disease, PDT+: tremor-dominant, PIGD: postural instability gait difficulty, SSD: Somatic symptom disorder.

## 4 Dopamine

DA is a monoamine neurotransmitter that modulates cognition, reward, motivation, learning, and the control of motor functions.^59^ A major cause of the characteristic motor symptoms of PD is the loss of dopaminergic neurons in the SNpc. The nigrostriatal pathway, located in the SNpc and associated with motor control, contains approximately 80% of the total dopaminergic neurons in the brain. Through this pathway, the projections of dopaminergic neurons reach the caudate and putamen regions of the basal ganglia in the dorsal striatum.^60^ The degeneration of nigrostriatal dopaminergic neurons results in an imbalance in the basal ganglia via the direct and indirect pathways, causing striatal DA deficiency and ultimately leading to bradykinesia.^61^ Moreover, DA deficiency in PD is not only a consequence of neuronal death but also results from impaired axonal DA release even prior to cell loss.^62^ DA exerts its effects by binding to G protein-coupled receptors after being released into the synaptic cleft.

DA receptors, which regulate physiological processes such as voluntary motor control and cognitive functions, are classified into two families, D1 and D2, with five subtypes in total.^63^ The D1-like family, including D_1_ receptors (D_1_Rs) and D_5_ receptors (D_5_Rs), is associated with stimulatory G proteins (Gs), whereas the D2-like family, including D_2_ receptors (D_2_Rs), D_3_ receptors (D_3_Rs), and D_4_ receptors (D_4_Rs), is linked to inhibitory G proteins (Gi and Go).^64^ In PD, changes in DA receptors occur due to dopaminergic neuronal loss and therapeutic interventions. In the early stages of PD, striatal D_2_R expression increases, but this expression declines approximately four years after the onset of motor symptoms.^65^

Because DA cannot cross the gastrointestinal mucosa or the blood–brain barrier (BBB), its lipophilic precursor L-DOPA is considered the gold standard for PD treatment. However, due to its short half-life, potential systemic side effects, and complications such as dyskinesia, alternative therapeutic approaches are being explored.^66^ One novel strategy involves continuous intracerebroventricular anaerobic DA (A-DA), which has shown high efficacy in improving motor dysfunction without inducing dyskinesia or tachyphylaxis in MPTP- and 6-OHDA-induced rat models.^67^ Clinically, the use of A-DA in PD treatment has also been investigated. A randomized controlled trial (Phase 1 and 2) examined the efficacy of intracerebroventricular A-DA in PD patients with L-DOPA-related complications. The treatment was delivered via a device-supported abdominal pump connected to a subcutaneous catheter implanted near the striatum into the third ventricle. Unlike L-DOPA, this approach did not trigger dyskinesia. These findings suggest that intracerebroventricular A-DA may represent a promising therapeutic option.^68^

An *in vivo* study developed a brain-targeted liposomal system (BTLS) using an amyloid precursor protein-derived peptide to facilitate effective DA transport across the BBB. This method enables the delivery of DA (normally unable to cross the BBB due to its polar nature) into the brain. The unique structure of BTLS allows rapid brain penetration and therapeutic efficacy at minimal DA doses. High-performance liquid chromatography (HPLC) analysis detected neither free DA nor its metabolites in peripheral circulation. Sustained and stable DA release into the brain could potentially reduce the risk of developing dyskinetic movements or neuropsychiatric complications such as psychosis. This method may offer a viable treatment for PD and other neurodegenerative diseases using low doses.^69^ Another *in vivo* study demonstrated that α-syn accumulation in dopaminergic terminals led to neuronal hyperexcitability and hypersensitivity in mice, contributing to PD pathology. In this model, modulation of D_2_R, which showed reduced expression in neurons with α-syn accumulation, was suggested as a therapeutic target.^70^ A clinical study using positron emission tomography (PET) imaging with the D_2_R/D_3_R ligand [18F] fallypride assessed striatal and extrastriatal D_2_R and D_3_R expression. It revealed that the severity of motor symptoms was positively correlated with D_2_R and D_3_R densities in the putamen and globus pallidus. Furthermore, abnormal D_2_R and D_3_R expression was observed in brain regions associated with both motor and nonmotor symptoms of PD. These findings suggest that D_2_R and D_3_R loss affects extrastriatal areas more significantly than basal ganglia regions, implicating both motor and nonmotor regions in disease progression.^71^

*In vivo* and *in vitro* studies explored blood biomarkers for early PD diagnosis in humans and experimental models. Findings indicated that reduced L-DOPA and dihydroxyphenylacetic acid (DOPAC) concentrations and increased *D3R* gene expression are specific to the preclinical phase of PD, making them suitable for early diagnosis.^72^ Elevated levels of 3,4-dihydroxyphenylacetaldehyde (DOPAL) (a highly reactive DA metabolite) have been linked to disrupted proteostasis and degeneration of neuronal projections via α-syn-related mechanisms in PD. DOPAL-induced α-syn accumulation impairs neuronal homeostasis, leading to dopaminergic neuron loss and motor dysfunction. Assessing DOPAL accumulation risk may be critical for early intervention in PD.^73^

Disruptions in neurotransmitter vesicle dynamics are also associated with neurodegenerative diseases like PD.^74^ In an MPTP-induced rat model, the expression of vesicular monoamine transporter 2 (VMAT2) was increased via a bacterial artificial chromosome (BAC). This upregulation improved DA release and mitigated neurodegeneration in PD. The observed increase in DA release and neuronal protection in VMAT2-overexpressing mice suggests that interventions aimed at enhancing vesicular capacity may offer therapeutic benefits in PD.^75^ Oxidative stress is another key contributor to neurodegeneration in PD.^76^ Glucose-6-phosphate dehydrogenase (G6PD), by generating nicotinamide adenine dinucleotide phosphate (NADPH), contribute to maintaining redox homeostasis; thus, its deficiency may exacerbate oxidative stress and promote neurodegeneration.^77^ Employing murine and human pluripotent stem cell PD models, along with human post-mortem tissue, investigated the role of G6PD deficiency in triggering DA loss and PD pathogenesis. Results showed that α-syn accumulation disrupts metabolic flux, reduces NADPH and glutathione (GSH) levels, promotes DA oxidation, and lowers the DA concentration. Clinical mutations in G6PD have been associated with PD diagnosis, and G6PD deletion has been implicated in PD pathology. Furthermore, genetic and pharmacological interventions to restore NADPH or GSH levels have been shown to prevent DA oxidation and rescue DA levels. These findings identify G6PD as a potential pharmacological target for PD treatment (Figure 1C).^78^

In summary, DA deficiency resulting from nigrostriatal degeneration underlies the motor and nonmotor symptoms of PD. Investigating DA metabolism, receptor dynamics, transport mechanisms, and interactions with other physiological systems may facilitate the discovery of both symptomatic and disease-modifying therapies for PD (Table 3).

**Table 3:** Summary of the Findings Reporting the Role of Dopamine in PD^a^

s.n.	study type	PD model	drug and dose	observation	remarks	refs
1	*In vitro*, *In vivo*	MPTP-induced C57Bl/6 J mice and 6-OHDA induced	A-DA (1 μL/h)	One novel strategy involves continuous intracerebroventricular A-DA, which has shown high efficacy in improving motor dysfunction without inducing dyskinesia or tachyphylaxis	A-DA, might be a new therapeutic strategy for PD	^67^
		Wistar rats, MPP-treated LUHMES cells				
2	*In vivo*	6-OHDA induced C57BL/6 mice, Sprague–Dawley rats, Sinclair mini-pigs	DA (800 μg/kg)	BTLS method enables sustained and stable DA release into the brain, this could potentially reduce the risk of developing dyskinetic movements or neuropsychiatric complications such as psychosis	HPLC analysis detected neither free DA nor its metabolites in peripheral circulation which means his method may offer a viable treatment for PD and other neurodegenerative diseases using low doses	^69^
3	*In vivo*	Wild-type C57BL/6J mice, DATIREScre and Ai95(RCL-GCaMP6f)-D (Ai95D) knock-in mice		α-syn accumulation in dopaminergic terminals leads to neuronal hyperexcitability and hypersensitivity in mice, contributing to PD pathology	Modulation of D_2_R, which showed reduced expression in neurons with α-syn accumulation, was suggested as a therapeutic target	^70^
4	*In vivo*	MPTP-induced mice		The expression of VMAT2 was increased via a BAC, this upregulation improved DA release and mitigated neurodegeneration in PD	The observed increase in DA release and neuronal protection in VMAT2-overexpressing mice suggests that interventions aimed at enhancing vesicular capacity may offer therapeutic benefits in PD	^75^
5	*In vitro*, *In vivo*, *Post-mortem Human study*	Murine and human pluripotent stem cell (hPSC) models of PD, Primary cortical neurons from E18 Sprague–Dawley rat embryos, SH-SY5Y-cells		G6PD deletion has been implicated in PD pathology, genetic and pharmacological interventions to restore NADPH or GSH levels were shown to prevent DA oxidation and rescue DA levels	These findings identify G6PD as a potential pharmacological target for PD treatment	^78^
		Homozygous M83 transgenic mice, B6C3F1 nontransgenic mice				
		Human post-mortem tissue				
6	*In vivo*, *Observational (Case-control) Human study*	MPTP-induced mice C57BL/6		Reduced L-DOPA and DOPAC concentrations and increased *D3R* gene expression are specific to the preclinical phase of PD, making them suitable for early diagnosis	DOPAL accumulation risk might be used for early PD diagnosis	^72^
		PD patients (*n* = 36), HC (*n* = 52)				
7	*Interventional Human study*	Phase 1: PD patients (*n* = 12)	A-DA (2, 10, 50, and 100 mg mL^–1^)	A-DA was delivered via a device-supported abdominal pump connected to a subcutaneous catheter implanted near the striatum into the third ventricle. Unlike L-DOPA, this approach did not trigger dyskinesia	Intracerebroventricular A-DA may represent a promising therapeutic option	^68^
		Phase 2: PD patients (*n* = 9)				
8	*Observational* (*Cross-sectional*) *Human study*	PD patients (*n* = 25), HC (*n* = 31)		The severity of motor symptoms are positively correlated with D_2_R and D_3_R density in the putamen and globus pallidus, abnormal D_2_R and D_3_R expression was observed in brain regions associated with both motor and nonmotor symptoms of PD	D_2_R and D_3_R loss affects extrastriatal areas more significantly than basal ganglia regions, implicating both motor and nonmotor regions in disease progression	^71^

a 6-OHDA: 6-hydroxydopamine, A-DA: anaerobic dopamine, BAC: bacterial artificial chromosome, BTLS: brain-targeted liposomal system, D2R: D2 receptor, D3R: D3 receptor, DA: dopamine, DOPAC: 3,4-dihydroxyphenylacetic acid, DOPAL: 3,4-dihydroxyphenylacetaldehyde, G6PD: glucose-6-phosphate dehydrogenase, GSH: glutathione, HPLC: high-performance liquid chromatography, L-DOPA: L-3,4-dihydroxyphenylalanine, MPTP: 1-methyl-4-phenyl-1,2,3,6-tetrahydropyridine, NADPH: nicotinamide adenine dinucleotide phosphate, PD: Parkinson’s disease, VMAT2: vesicular monoamine transporter 2, α-syn: α-synuclein.

## 5 Serotonin

Serotonin (5-HT) is a monoamine neurotransmitter found not only in the CNS but also in peripheral tissues.^79^ In the CNS, 5-HT is produced by neurons located in the raphe nuclei of the brain and is involved in the regulation of behavioral functions such as mood, perception, memory, and stress. In the peripheral nervous system (PNS), 5-HT modulates physiological functions including heart rate, gastrointestinal motility, and vasoconstriction.^79,80^ The synthesis of 5-HT occurs through the hydroxylation of tryptophan to 5-hydroxytryptophan (5-HTP) by tryptophan hydroxylase (TPH), followed by decarboxylation of 5-HTP.^81^ In the brain, 5-HT is synthesized by tryptophan hydroxylase 2 (TPH2), while in the gut, it is synthesized by tryptophan hydroxylase 1 (TPH1), particularly by enterochromaffin cells, which constitute the primary source of peripheral 5-HT.^82^

5-HT exerts its effects in the CNS and PNS through receptors classified into seven families (5-HT1–7) based on their signaling mechanisms.^83^ While 5-HT1, 2, and 5-HT4–7 receptors belong to the G protein-coupled receptor family, 5-HT3 is a ligand-gated ion channel. The diversity of 5-HT receptors allows for a more nuanced understanding of serotonergic system functions.^84^ In addition to receptors, 5-HT transporters (SERT) are crucial components of the serotonergic system. SERT terminates the action of 5-HT via sodium (Na^+^)- and Cl^–^-dependent reuptake into the presynaptic neuron. As such, SERT is an important pharmacological target due to its role in extending 5-HT’s action within the synaptic cleft.^85^ Dysregulation of the serotonergic system, involving both SERT and 5-HT receptors, contributes to the pathogenesis of motor and nonmotor symptoms of PD.^86^

Agonists of 5-HT1A receptors have been proposed to reduce the LID in PD animal models. The biased presynaptic 5-HT1A agonist F13714 demonstrated potent antidyskinetic activity and nearly completely suppressed abnormal involuntary movements associated with LID. There is a need to develop selective, full agonists of 5-HT receptors for treating PD patients suffering from peak-dose LID.^87^ Another study investigating the role of the serotonergic system in LID demonstrated that NLX-112, a biased 5-HT1A agonist, exerted strong anticyskinetic effects in hemiparkinsonian rats. Neuroimaging results showed that NLX-112 prevented LID by activating presynaptic autoreceptors in the raphe nuclei. Thus, targeting 5-HT1A receptors may offer a promising therapeutic strategy for LID.^88^

Working memory impairment, a common symptom in PD, has been associated with dysfunction in the medial septum-diagonal band (MS-DB) complex and the modulation of 5-HT6 receptors. An *in vivo* study on unilateral 6-OHDA-lesioned PD rats revealed that both activation and blockade of 5-HT6 receptors in the MS-DB affected working memory by increasing DA and NE levels in the mPFC and hippocampus. These findings suggest that 5-HT6 receptors may serve as therapeutic targets for addressing cognitive and learning deficits in PD.^89^

An observational study involving PD patients, subjects with possible PD but no evidence of dopaminergic deficiency (SWEDD individuals), and HCs examined the correlation between early serotonergic raphe dysfunction and both motor and nonmotor symptoms in PD. Only approximately 13% of PD patients exhibited raphe serotonergic involvement. Reduced raphe SERT availability was correlated with the severity of resting tremor but not with nonmotor symptoms such as fatigue, depression, or sleep disturbances. This implies that while serotonergic pathways could be targeted in advanced PD, 5-HT may not be the principal target for treating nonmotor symptoms like depression.^90^ While SERT levels decrease in late-stage PD, evidence suggests that SERT is upregulated in dyskinetic and LID-afflicted rats. Genetic knockout of SERT in hemiparkinsonian rats reduced LID without diminishing the motor benefits of L-DOPA, indicating that SERT-targeted adaptations could be effective for LID management.^91^ In a MitoPark rat model of PD, 5-HT precursor 5-HTP and SERT inhibitor citalopram were found to mitigate LID symptoms. Interestingly, 5-HTP and citalopram suppressed different subtypes of abnormal involuntary movements: citalopram primarily reduced axial movements, whereas 5-HTP targeted limb movements. These differential effects may relate to the drugs’ impact on L-DOPA-derived DA and 5-HT metabolism.^92^

Given 5-HT’s role in sleep and arousal regulation, its involvement in sleep disorders, a common nonmotor symptom in PD, has been investigated. Promising imaging studies using [^11^C]­DASB PET to visualize SERT showed reduced serotonergic activity in the midbrain raphe, basal ganglia, and hypothalamus of both humans and animals. This suggests that therapeutic strategies aiming to enhance 5-HT levels may improve sleep dysfunctions in PD.^93^

Apathy, often accompanied by anxiety and depression, is among the most frequent neuropsychiatric disorders in PD. In *de novo* PD patients, serotonergic dysfunction, not dopaminergic degeneration, was found to be the primary determinant of apathy, depression, and anxiety. This points to a potential role of serotonergic degeneration in the pathogenesis of neuropsychiatric symptoms during early PD.^94^

In a BAC α-syn transgenic rat model of PD, early serotonergic deficits were linked to impaired hippocampal neurogenesis. Notably, serotonergic dysfunction in the hippocampus preceded motor symptoms and was associated with α-syn aggregation and reduced 5-HT levels. These findings suggest a serotonergic contribution to the pathogenesis of nonmotor symptoms, such as depression and anxiety, during the premotor stages of PD.^95^

A dissection study on human post-mortem hemispheres explored the serotonergic system through high-resolution population-based tractography and previously identified deep brain stimulation (DBS) hotspots. The study revealed that the ventral tegmental area (VTA) is part of an extensive network involving serotonergic pontine nuclei. Targeted modulation of specific VTA connections, while avoiding projections to regions such as the lateral hypothalamus (implicated in autonomic cardiac side effects), may facilitate the development of personalized DBS strategies (Figure 1D).^96^

In summary, 5-HT has been implicated in early-stage raphe serotonergic dysfunction and is associated with resting tremors and neuropsychiatric symptoms in PD. Disruption of the serotonergic system is linked to apathy, depression, sleep disturbances, and cognitive deficits. Early identification of serotonergic degeneration may contribute to the development of novel therapeutic strategies for PD management (Table 4).

**Table 4:** Summary of the Findings Reporting the Role of Serotonin in PD^a^

s. n.	study type	PD model	drug and dose	observation	remarks	references
1	*In vivo*	6-OHDA Sprague–Dawley rats	l-DOPA/Benserazide (6/12 mg/kg)	The biased presynaptic 5-HT1A agonist F13714 demonstrated potent antidyskinetic activity and nearly completely suppressed abnormal involuntary movements associated with LID.	There is a need to develop selective, full agonists of 5-HT receptors for treating PD patients suffering from peak-dose LID.	^87^
			F13714 (0.02–0.04 mg/kg)			
2	*In vivo*	6-OHDA Sprague–Dawley rats	L-DOPA/benserazide (6/12 mg/kg)	Neuroimaging results showed that NLX-112 prevented LID by activating presynaptic autoreceptors in the raphe nuclei.	Thus, targeting 5-HT1A receptors may offer a promising therapeutic strategy for LID.	^88^
		HPK-non-LID rats	NLX-112 (0.16 mg/kg)			
3	*In vivo*	6-OHDA mice	WAY208466 (3, 6, and 12 μg/rat)	An *in vivo* study on unilateral 6-OHDA-lesioned PD rats revealed that both activation and blockade of 5-HT6 receptors in the MS-DB affected working memory by increasing dopamine and norepinephrine levels in the mPFC and hippocampus.	These findings suggest that 5-HT6 receptors may serve as therapeutic targets for addressing cognitive and learning deficits in PD.	^89^
			SB258585 (2, 4, and 8 μg/rat)			
4	*In vivo*	Sprague–Dawley rats		Genetic knockout of SERT in hemiparkinsonian rats reduced LID without diminishing the motor benefits of L-DOPA, indicating that SERT-targeted adaptations could be effective for LID management	Adaptations targeting SERT may be effective in treating LID.	^91^
5	*In vivo*	MitoPark mice	5-HTP (50 mg/kg)/carbidopa (5 mg/kg)	The 5-HT precursor 5-HTP and the SERT inhibitor citalopram have been found to attenuate the development of LID. 5-HTP and citalopram suppressed different subtypes of abnormal involuntary movements: citalopram primarily reduced axial movements, whereas 5-HTP targeted limb movements.	These differential effects may relate to the drugs’ impact on L-DOPA-derived dopamine and 5-HT metabolism.	^92^
			Citalopram (50 mg/kg)			
6	*In vivo*	BAC transgenic rat model		Serotonergic dysfunction in the hippocampus preceded motor symptoms and was associated with α-synuclein aggregation and reduced 5-HT levels	These findings suggest a serotonergic contribution to the pathogenesis of nonmotor symptoms, such as depression and anxiety, during the premotor stages of PD.	^95^
7	*Observational Human Study*	PD (*n* = 345)		Reduced raphe SERT availability was correlated with the severity of resting tremor but not with nonmotor symptoms such as fatigue, depression, or sleep disturbances.	While serotonergic pathways could be targeted in PD+, serotonin may not be the principal target for treating nonmotor symptoms like depression.	^90^
		HC (*n* = 185)				
		SWEDD (*n* = 56)				
8	*Observational Human Study*	PD patients with sleep dysfunction (*n* = 14)		Promising imaging studies using [^11^C]DASB PET to visualize SERT showed reduced serotonergic activity in the midbrain raphe, basal ganglia, and hypothalamus of both humans and animals.	Therapeutic strategies aiming to enhance 5-HT levels may improve sleep dysfunctions in PD.	^93^
		PD patients without sleep dysfunction (*n* = 14)				
		HC (*n* = 12)				
9	*Observational Human Study*	Apathetic PD (*n* = 15)		In de novo PD patients, serotonergic dysfunction, not dopaminergic degeneration, was found to be the primary determinant of apathy, depression, and anxiety	This points to a potential role of serotonergic degeneration in the pathogenesis of neuropsychiatric symptoms during early PD.	^94^
		Nonapathetic PD (*n* = 15)				
		HC (*n* = 15)				
10	*Post-mortem Human Study*			Using population-based high-resolution fiber tractography and previously reported DBS hotspots, the VTA has been shown to participate in a widespread network involving serotonergic pontine nuclei.	Targeted modulation of specific VTA connections, while avoiding projections to regions such as the lateral hypothalamus (implicated in autonomic cardiac side effects), may facilitate the development of personalized DBS strategies.	^96^

a 5-HT: serotonin, 5-HTP: 5-hydroxytryptophan, BAC: bacterial artificial chromosome, DBS: deep brain stimulation, HPK: hemiparkinsonizm, LID: L-DOPA-induced dyskinesia, mPFC: medial prefrontal cortex, MS-DB: medial septum-diagonal band, NE: norepinephrine transporter, PD: Parkinson’s Disease, SERT: serotonin transporter, SWEDD: subjects with possible PD but no evidence of dopaminergic deficiency, PET: positron emission tomography, VTA: ventral tegmental area, α-syn: α-synuclein.

## 6 Histamine

Histamine is a monoamine neurotransmitter that contributes to the regulation of fundamental bodily functions, including the sleep-wake cycle, energy-endocrine homeostasis, synaptic plasticity, and learning.^97^ In the adult mammalian brain, the primary source of histamine is the tuberomammillary nucleus (TMN) located in the posterior hypothalamus.^98^ Interactions between histamine and other neurotransmitter systems form a network that links basic homeostatic mechanisms with higher brain functions such as learning and memory.^97^

Histamine exerts its effects via four G protein-coupled receptors: H1R, H2R, H3R, and H4R.^99^ While H1R, H2R, and H3R are primarily expressed in the brain, H4R is found peripherally.^97^ Postsynaptic H1R and H2R typically elicit excitatory responses.^100^ H3R, which is densely localized in histaminergic neurons, functions as an autoreceptor and modulates the release of histamine and other neurotransmitters such as GABA, glutamate, acetylcholine (ACh), and norepinephrine (NE).^101^ Activation of H3R leads to autoinhibition of TMN neurons and suppression of histamine release and synthesis.^102^ Abundantly expressed in the striatum, H3R has been shown to influence DA release, and one study demonstrated that postsynaptic H3R significantly contributes to the negative modulation of D2 receptors.^103^

An *in vivo* study investigated the potential effects of histamine on dopaminergic neurons via H1R. The results revealed that histamine induces microglial phagocytosis through H1R activation and triggers reactive oxygen species (ROS) production via H1R and H4R. Furthermore, the H1R blockade was found to provide protection against *in vivo* dopaminergic neuron loss. H1R antagonists may thus represent promising agents for the treatment of PD and other neurodegenerative disorders.^104^ Another *in vivo* study targeting histamine receptors in the entopeduncular nucleus (EPN)–thalamus circuit aimed to alleviate motor dysfunction in PD. The findings showed that increased histaminergic innervation in the EPN activated parvalbumin-expressing EPN neurons projecting to motor thalamic nuclei via postsynaptic H2R-mediated hyperpolarization through hyperpolarization-activated cyclic nucleotide-gated channels (HCN). Simultaneously, this effect was negatively regulated by presynaptic H3R activation in glutamatergic neurons projecting from the subthalamic nucleus (STN) to the EPN. Notably, activation of both receptor types improved motor impairments observed in PD. Thus, targeting H2R and H3R in the EPN-thalamus circuit holds therapeutic promise for treating motor dysfunction in PD.^105^

In a 6-OHDA-induced rat model of PD, the H3R antagonist thioperamide was shown to preserve the circadian rhythm and memory functions. The treatment normalized rest/activity cycles and ameliorated recognition memory deficits associated with PD. However, thioperamide did not exhibit a significant effect on anxiety-like behaviors induced by 6-OHDA. These results suggest that thioperamide may offer therapeutic potential for treating cognitive deficits and circadian rhythm disturbances in PD.^106^

A post-mortem study investigated alterations in the histaminergic system within the substantia nigra and striatum of PD patients. A significant reduction in H3R mRNA expression was observed in the substantia nigra, while H4R mRNA expression—typically limited in the brain, was found to be increased in the caudate nucleus and putamen. Additionally, elevated mRNA levels of histamine *N*-methyltransferase (HMT), the enzyme responsible for histamine inactivation via methylation, were detected in both the substantia nigra and putamen. These findings suggest that local changes in the histaminergic system may contribute to the pathophysiology of PD.^107^ In a complementary *in vitro* and *in vivo* study, targeting H4R was explored for its potential therapeutic role in PD. The H4R antagonist JNJ7777120 was shown to inhibit pro-inflammatory microglia and prevent the progression of PD pathology in a rotenone-induced rat model. This included prevention of dopaminergic neurodegeneration and striatal DA depletion as well as a reduction in LB-like pathology. H4R may thus represent a promising target for combating microglial activation and PD progression.^108^

Consistent with these findings, another *in vivo* study showed that JNJ7777120 normalized the number of nigrostriatal dopaminergic fibers and striatal DA levels in a rotenone-induced PD rat model. The compound also restored basal ganglia function by reducing striatal GABA levels and the 5-hydroxyindoleacetic acid (5-HIAA)/5-HT ratio. These findings highlight the importance of targeting microglial H4R as a promising and specific therapeutic strategy to mitigate neuroinflammation and disease progression in PD.^109^

In a post-mortem human study, despite the accumulation of LB and Lewy neurites (LN) in the TMN of PD patients, neuronal histamine production was found to remain unchanged. Assessments of histidine decarboxylase (HDC) mRNA levels—the enzyme responsible for histamine synthesis—and the quantity of LB/LN in the TMN revealed no significant differences across clinical or Braak stages of PD.^110^ Another study using a 6-OHDA-induced rat model of PD examined the effects of histaminergic innervation of the ventral anterior thalamic nucleus (VA), a region receiving direct input from the hypothalamic histaminergic system and showing reduced activity in PD. Results indicated that histaminergic afferents modulate VA activity to actively compensate for motor deficits associated with PD. Thus, targeting VA histamine receptors and downstream ion channels could represent a potential therapeutic strategy for PD-related motor dysfunction (Figure 2B).^111^

**Figure 2:**
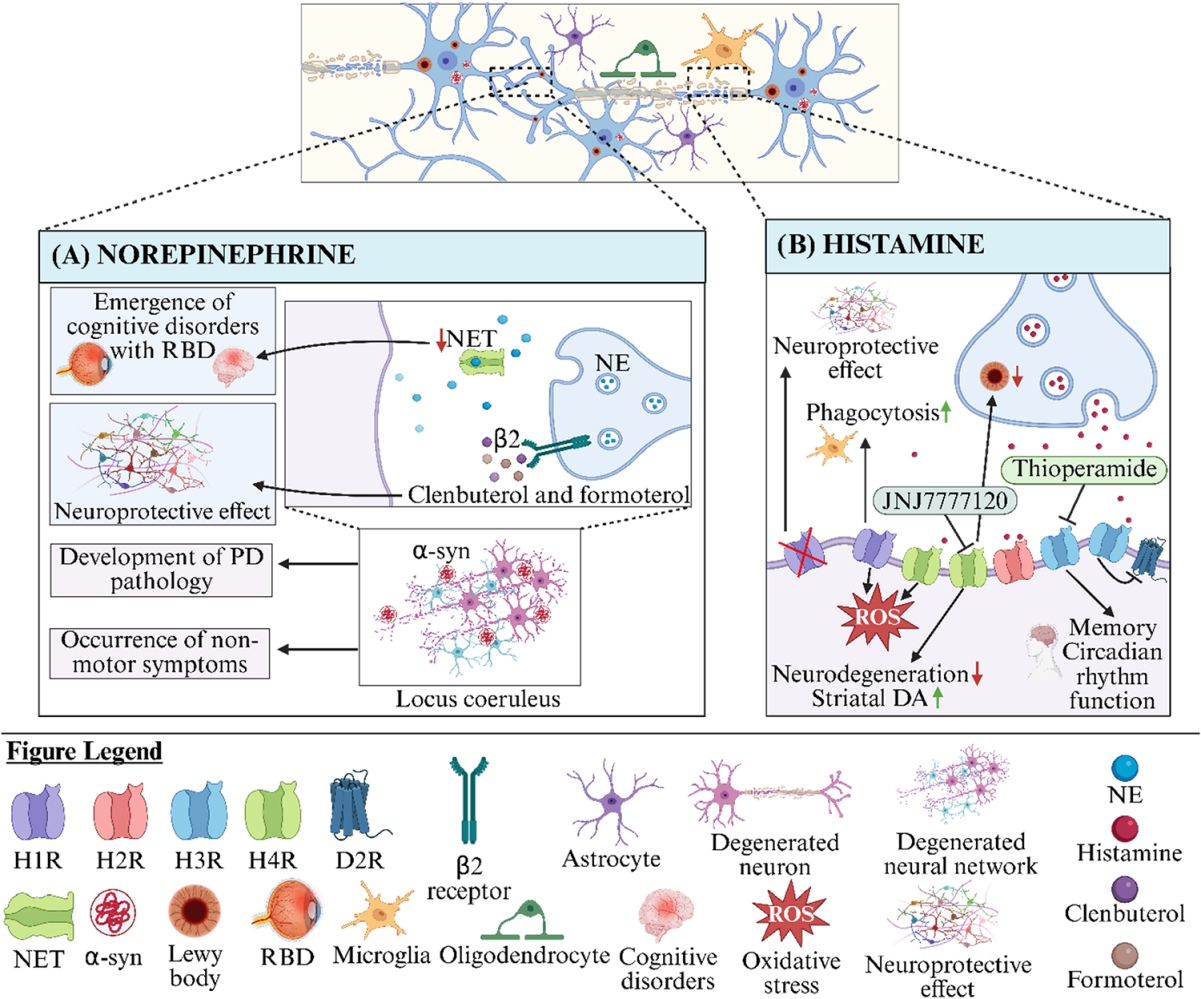
(A) The role of NE in PD. β2-adrenergic receptor activation modulates neuroinflammation in vivo. β2-adrenergic receptor may also have neuroprotective properties in conditions where inflammation promotes dopaminergic neurodegeneration.^128^ A study using PET imaging and neuromelanin-sensitive MRI with the selective NET radioligand 11C-MeNER has shown that noradrenergic dysfunction in PD is associated with RBD.^124^ α-Syn pathology affects LC neurons, and noradrenergic dysfunction contributes to early PD pathogenesis.^126^ (B) The role of histamine in PD. Histamine stimulates microglial phagocytosis by activating H1 receptors and increases ROS production through both H1R and H4R. Furthermore, blockade of H1R has been shown to protect against the loss of dopaminergic neurons in vivo.^104^ The H3R antagonist thioperamide has been found to be effective in maintaining circadian rhythm and memory functions.^106^ The H4R antagonist JNJ7777120 has been shown to halt the progression of PD pathology by suppressing the activity of pro-inflammatory microglia. These effects were observed in combination with the prevention of dopaminergic neuron loss and striatal dopamine depletion, as well as a reduction in LB-like pathology.^108^ *D*_2_ *R: D*_2_ *receptor*, *H1R: histamine receptor 1*, *H2R: histamine receptor 2*, *H3R: histamine receptor 3*, *H4R: histamine receptor 4*, *NE: Norepinephrine*, *NET: Norepinephrine transporter*, *RBD*: *Rapid eye movement sleep behavior disorder*, *α-syn: α-synuclein*.

Targeting histaminergic system receptors has demonstrated neuroprotective and memory-preserving effects. Furthermore, studying histamine receptor expression patterns can enhance our understanding of the role of the histaminergic system in the brain during PD. Exploring the relationship between histamine and other neurotransmitter systems, receptor-specific interventions, and genetic factors influencing the histaminergic system may pave the way for its use as a therapeutic target in PD (Table 5).

**Table 5:** Summary of the Findings Reporting the Role of Histamine in PD^a^

s.n.	study type	PD model	drug and dose	observation	remarks	refs
1	*In vivo*, *in vitro*	Murine N9 microglial cell line, Murine primary microglial cell cultures, Wild-type C57BL/6 mice	Mepyramine maleate, (1 μM), JNJ777712 (5 μM)	Histamine induces microglial phagocytosis through H1R activation and triggers ROS production via H1R and H4R, H1R blockade was found to provide protection against *in vivo* dopaminergic neuron loss	H1R antagonists may thus represent promising agents for the treatment of PD and other neurodegenerative disorders	^104^
2	*In vivo*, *in vitro*	Rotenone-induced PD Sprague–Dawley rats, Rotenone-induced human SH-SY5Y neuroblastoma cell	JNJ7777120 (ICV, 5 μg/day)	The H4R antagonist JNJ7777120 was shown to inhibit pro-inflammatory microglia and prevent the progression of PD pathology by preventing dopaminergic neurodegeneration and striatal DA depletion, as well as reducing LB-like pathology	H4R may thus represent a promising target for combating microglial activation and PD progression	^108^
3	*In vivo*	6-OHDA induced B6/JGpt-Rosa26^tm1 Cin(CAG‑LSL‑Cas9‑tdTomato)^/Gpt mice, B6/JGptPvalbemlCin(IRES-iCre)/Gpt mice, C57BL/6-Slc17a6^em1(IRESiCre)Smoc^, wild-type (WT) C57BL/6J mice	Histamine (1–30 μM)	Increased histaminergic innervation in the EPN activated parvalbumin-expressing EPN neurons projecting to motor thalamic nuclei via postsynaptic H2R-mediated hyperpolarization through HCN, this effect was negatively regulated by presynaptic H3R activation in glutamatergic neurons projecting from the STN to the EPN	Activation of both receptor types improved motor impairments observed in PD, this may mean that targeting H2R and H3R in the EPN-thalamus circuit holds therapeutic promise for treating motor dysfunction in PD	^105^
			Dimaprit (10–100 μM)			
			RAMH (10 μM)			
			Ranitidine (0. 3–1 μM)			
			IPP (1 μM)			
			ZD7288 (10 μM)			
4	*In vivo*	6-OHDA induced C57BL/6N mice	Thioperamide (20 mg kg^–1^)	Thioperamide was shown to preserve circadian rhythm and memory functions, it normalized rest/activity cycles and ameliorated recognition memory deficits associated with PD	Thioperamide may offer therapeutic potential for treating cognitive deficits and circadian rhythm disturbances in PD	^106^
5	*In vivo*, *Post-mortem Human study*	Rotenone-induced Sprague–Dawley rats, post-mortem PD patients and HC’s	JNJ7777120 (5 μg/day)	JNJ7777120 normalized the number of nigrostriatal dopaminergic fibers and striatal DA levels, restored basal ganglia function by reducing striatal GABA levels and the 5-HIAA/5-HT ratio	These findings highlight the importance of targeting microglial H4R as a promising and specific therapeutic strategy to mitigate neuroinflammation and disease progression in PD	^109^
6	*In vivo*, *Observational Human study*	6-OHDA induced Sprague–Dawley rats, transgenic rat strain expressing Cre recombinase in HDC neurons	Mepyramine (3.5 μg) or Ranitidine (4 μg)	Histaminergic afferents modulate VA activity to actively compensate for motor deficits associated with PD	Targeting VA histamine receptors and downstream ion channels could represent a potential therapeutic strategy for PD-related motor dysfunction	^111^
		PD patients (*n* = 22), HC (*n* = 21)				
7	*Post-mortem Human study*	Post-mortem brain samples of PD (*n* = 7) and HC (*n* = 7) samples		A significant reduction in H3R mRNA expression was observed in the substantia nigra, while H4R mRNA expression—typically limited in the brain—was found to be increased in the caudate nucleus and putamen; elevated mRNA levels of HMT were detected in both the substantia nigra and putamen	Local changes in the histaminergic system may contribute to the pathophysiology of PD	^107^
8	*Post-mortem Human study*	PD patients with different stages of PD (*n* = 15), HC (*n* = 15)		Despite the accumulation of LB and LN in the TMN of PD patients, neuronal histamine production was found to remain unchanged	Assessments of HDC mRNA levels and the quantity of LB/LN in the TMN revealed no significant differences across clinical or Braak stages of PD	^110^

a 5-HIAA: 5-hydroxyindoleacetic acid, 5-HT: serotonin, 6-OHDA: 6-hydroxydopamine, DA: dopamine, EPN: entopeduncular nucleus, GABA: γ-aminobutyric acid, HDC: histidine decarboxylase, HCN: hyperpolarization-activated cyclic nucleotide-gated channels, HMT: histamine *N*-methyltransferase, IPP: iodophenpropit, LB: Lewy body, LN: Lewy neurites, PD: Parkinson’s disease, RAMH: R-α-methylhistamine, ROS: reactive oxygen species, STN: subthalamic nucleus, TMN: tuberomammillary nucleus, VA: ventral anterior thalamic nucleus.

## 7 Norepinephrine

NE, also known as noradrenaline, is a monoamine neurotransmitter primarily released from the locus coeruleus (LC) in the brain.^112^ The LC-NE system exerts extensive innervation throughout the CNS, regulating a wide range of behaviors including arousal, attention, working memory, sensory processing, and the interaction between reward and attention.^113,114^ This broad spectrum of functions is closely related to the synthesis and release mechanisms of NE within LC neurons.

In LC neurons, NE is synthesized via the conversion of DA—produced from tyrosine by the enzyme tyrosine hydroxylase—into NE by DA-β-hydroxylase.^115^ NE exerts its synaptic effects by binding to NE receptors after being transported back into presynaptic neurons through NE transporters (NET).^116^ NE receptors belong to three families of G protein-coupled receptors: α1, α2, and β. While α1 and β receptors are mainly located postsynaptically and mediate excitatory effects, α2 receptors are found both pre- and postsynaptically and are involved in inhibitory modulation.^115,117^

Reduced synthesis or action of NE, associated with the degeneration of LC and sympathetic ganglion neurons, is considered a component of neurodegenerative processes such as PD.^118,119^ Imaging of the LC has been evaluated as a potential biomarker for noradrenergic dysfunction in neurodegenerative diseases like PD.^120^ *In vivo* evidence of noradrenergic deficits in PD patients has been demonstrated using PET imaging with the selective NET radioligand ^11^C-MeNER. In PD patients, both LC degeneration and reduced NET density are indicators of noradrenergic involvement in the disease process, particularly concerning the emergence of nonmotor symptoms.^121^ Another in vivo human study utilizing ^11^C-MeNER PET aimed to quantify the reduced NET density in the motor cortex of PD patients. The results provided *in vivo* evidence of impaired noradrenergic function in the primary motor cortex correlating with disease severity, suggesting that noradrenergic dysfunction may contribute to motor symptoms beyond dopaminergic degeneration.^122^

The association between regional LC degeneration and noradrenergic neuron loss in PD has also been investigated. A multimodal *in vivo* imaging study involving HCs and PD patients assessed noradrenergic terminals using ^11^C-MeNER PET (selective for NET) and somatic cell bodies using turbo spin–echo magnetic resonance imaging (TSE MRI) (sensitive to neuromelanin content). The findings revealed that axonal damage was more pronounced than somatic damage, indicating differential vulnerability within the noradrenergic system to neurodegeneration in PD.^123^

A separate neuroimaging study using ^11^C-MeNER PET and neuromelanin-sensitive MRI also demonstrated that noradrenergic dysfunction in PD is associated with rapid eye movement sleep behavior disorder (RBD), further linking noradrenergic alterations to nonmotor symptoms. The presence of RBD in PD patients may contribute to cognitive decline.^124^ In another multimodal *in vivo* imaging study, neuromelanin-sensitive MRI assessed LC pigmented neurons, and ^11^C-yohimbine PET evaluated central α2-adrenergic receptor density in both PD and HC groups. PD patients exhibited a reduced neuromelanin signal in the LC and decreased ^11^C-yohimbine binding in both the motor cortex and widespread cortical regions. These results encourage further exploration of α2-adrenergic receptor-targeted therapies for PD treatment.^125^

A transgenic mouse model expressing wild-type α-syn in noradrenergic neurons revealed a relationship between LC pathology and the emergence of nonmotor symptoms in PD. The study demonstrated that α-syn pathology affects LC neurons and that noradrenergic dysfunction contributes to early PD pathogenesis. LC degeneration may represent a turning point in PD progression.^126^

Interestingly, preserved noradrenergic function has been observed in PD patients with resting tremor. Despite significant losses in NE terminal function across PD patients (with or without tremor) compared with HCs, relatively intact noradrenergic neurons were found in the LC and thalamus of patients with PDT+. A larger longitudinal study could further clarify the role of NE in tremor modulation.^127^ In a pharmacological study targeting β2-adrenergic receptors, a subtype of NE β-receptors, a lipopolysaccharide (LPS) inflammatory rat model of PD was used. The results indicated that β2-adrenergic receptor activation modulated *in vivo* neuroinflammation, suggesting a neuroprotective potential in conditions where inflammation promotes dopaminergic neurodegeneration. Thus, β2-adrenergic receptor agonists may offer disease-modifying effects through immunomodulation.^128^

In addition to changes in NE levels, the noradrenergic system dysfunction may contribute to PD pathogenesis via effects on the dopaminergic system. An *in vivo* study on DBH:BDNF transgenic mice using the neurotoxin DSP-4 showed that noradrenergic input is necessary for the survival of vulnerable midbrain dopaminergic (mDA) neurons in neurodegenerative diseases such as PD and AD. The researchers proposed a mechanism whereby LC afferents provide anterograde trophic support through brain-derived neurotrophic factor (BDNF) and NE activity, thereby promoting mDA neuron survival.^129^ Similarly, another animal study demonstrated that an intact LC supports the maintenance of nigral dopaminergic neurons and motor function, suggesting early loss of such trophic support may contribute to PD pathogenesis (Figure 2A).^130^

In conclusion, dysfunctions in the noradrenergic system—through LC degeneration, alterations in NE levels, and interactions with the dopaminergic system—contribute to both motor and nonmotor symptoms in PD. Noradrenergic-targeted therapeutic strategies hold promise for slowing disease progression (Table 6).

**Table 6:** Summary of the Findings Reporting the Role of Norepinephrine in PD^a^

s. n.	study type	PD model	drug and dose	observation	remarks	references
1	*In vivo*	DBH-hSNCA mice on C57BL/6 background		The study demonstrated that α-Syn pathology affects LC neurons and that noradrenergic dysfunction contributes to early PD pathogenesis	LC degeneration may represent a turning point in PD progression.	^126^
2	*In vivo*	Wistar rats	Clenbuterol (100 μg-kg^–1^), formoterol (100 μg-kg^–1^)	The results indicated that β2-adrenergic receptor activation modulated *in vivo* neuroinflammation, suggesting a neuroprotective potential in conditions where inflammation promotes dopaminergic neurodegeneration.	β2-adrenergic receptor agonists may offer disease-modifying effects through immunomodulation.	^128^
3	*In vivo*	DBH:BDNF transgenic mice and Sprague–Dawley rats	DSP-4; Sigma, (50 mg/kg)	The researchers proposed a mechanism whereby LC afferents provide anterograde trophic support through BDNF and NE activity, thereby promoting mDA neuron survival.	Early loss of NE activity and anterograde neurotrophin support may contribute to the degeneration of vulnerable neurons in neurodegenerative diseases such as PD.	^129^
		Wild-type littermates				
4	*In vivo*	Sprague–Dawley rats	DSP-4 (50 mg/kg)	An intact LC supports the maintenance of nigral dopaminergic neurons and motor function.	Early loss of anterograde trophic support, which is also mentioned in the pathogenesis of PD, may be the case.	^129^
5	*In vivo*, *Human Study*	PD patient (*n* = 15)		*In vivo* evidence of noradrenergic deficits in PD patients has been demonstrated using PET imaging with the selective NET radioligand ^11^C-MeNER.	In PD patients, both LC degeneration and reduced NET density are indicators of noradrenergic involvement in the disease process, particularly concerning the emergence of nonmotor symptoms.	^121^
		HC (*n* = 10)				
6	*In vivo*, *Human Study*	PD patient (*n* = 30)		*In vivo* evidence of impaired noradrenergic function in the primary motor cortex has been reported using 11C-MeNER PET measurement, which correlates with PD severity.	Noradrenergic dysfunction may contribute to motor symptoms beyond dopaminergic degeneration.	^122^
		HC (*n* = 12)				
7	*Human Study*	PD patient (*n* = 53)		Axonal damage was more pronounced than somatic damage, indicating differential vulnerability within the noradrenergic system to neurodegeneration in PD.	The study findings may guide research into potential new treatment strategies that specifically target the most affected structures.	^123^
		HC (*n* = 40)				
8	*In vivo*, *Human Study*	PD patient (*N* = 30)		The study using 11C-MeNER PET and neuromelanin-sensitive MRI demonstrated that noradrenergic dysfunction in PD is associated with rapid eye movement sleep behavior disorder (RBD), further linking noradrenergic alterations to nonmotor symptoms.	The presence of RBD in PD patients may contribute to cognitive decline.	^124^
		16 patients with RBD and 14 without RBD				
		HC (*n* = 12)				
9	*In vivo*, *Human Study*	PD patient (*n* = 30)		PD patients exhibited reduced neuromelanin signal in the LC and decreased ^11^C-yohimbine binding in both the motor cortex and widespread cortical regions.	These results encourage further exploration of α2-adrenergic receptor-targeted therapies for PD treatment.	^125^
		HC (*n* = 30)				
10	*Human Study*	PD patient (*n* = 65)		Despite significant losses in NE terminal function across PD patients (with or without tremor) compared to HCs, relatively intact noradrenergic neurons were found in the LC and thalamus of patients with tremor (PDT+).	It has been demonstrated that noradrenergic neurons in the LC and thalamus are relatively preserved in PDT+.	^125^
		HC (*n* = 28)				

a BDNF: brain-derived neurotrophic factor, DSP-4: N-(2-chloroethyl)-N-ethyl-2-bromobenzylamine, HC: healthy controls, LC: locus courelus, mDA: midbrain dopaminergic, MRI: magnetic resonance imaging, NE: norepinephrine, NET: norepinephrine, PD: Parkinson’s Disease, PDT+: tremor-dominant, PDT–: tremor absent, PET: positron emission tomography, RBD: rapid eye movement sleep behavior disorder.

## 8 Acetylcholine

ACh is a neurotransmitter synthesized by the enzymatic reaction of choline and acetyl-CoA catalyzed by choline acetyltransferase (ChAT).^131,132^ ACh receptors are broadly classified into two major types: nicotinic (nAChRs), which mediate fast synaptic transmission, and muscarinic (mAChRs), which are responsible for slower metabolic responses.^133^ Ionotropic nAChRs, which form ligand-gated excitatory ion channels permeable to Na+, potassium (K^+^), and calcium (Ca^2+^), are composed of various subunits such as α, β, δ, ε, and γ in distinct pentameric combinations.^134^ These receptors are implicated in numerous physiological and pathological processes including synaptic transmission, modulation of neurotransmitter release, neuropathic pain, inflammation, and cancer.^135^

mAChRs, which are G protein-coupled, are subdivided into five subtypes: M1R, M2R, M3R, M4R, and M5R.^136^ M1R, M3R, and M5Rs couple with the Gq/11 protein family, while M2R and M4Rs couple with the Gi/o family.^137^ A study in mice demonstrated that endothelial M3R deficiency leads to reduced vascular reactivity, increased blood pressure, and impaired cognitive functions.^138^ Another component of the cholinergic system, the vesicular ACh transporter (VAChT), is a protein responsible for packaging and transporting ACh for exocytotic release. VAChT dysfunction has been associated with neurological disorders such as PD and congenital myasthenic syndromes.^139,140^

In PD, heterogeneous alterations in the cholinergic system occur across various brain regions.^141^ Striatal cholinergic interneurons, which contribute to the striatal circuitry involved in reward and associated behaviors, can be adversely affected by conditions with impaired dopaminergic signaling such as PD.^142^ An *in vivo* study demonstrated that ACh release in the mouse striatum exhibits oscillatory activity similar to DA, and the spatial scale of striatal DA release is expanded by nAChRs.^143^ In another *in vivo* study, the absence of α5 nAChR in hemiparkinsonian mice was associated with reduced dopaminergic neurodegeneration and motor dysfunction, suggesting that nAChRs containing the α5 subunit may represent novel therapeutic targets for PD.^144^

In a 6-OHDA-induced rat model of PD, striatal and nigral M1Rs and M4Rs have been shown to modulate LID and GABAergic activation of the striato-nigral pathway. The findings indicate that striatal M1Rs facilitate *in vivo* dyskinesia and activation of the striato-nigral pathway, whereas striatal M4Rs may either facilitate or inhibit dyskinesia depending on their localization.^145^ Traditional theories propose that pathological increases in cholinergic signaling contribute to elevated ACh release, thereby exacerbating motor deficits in PD. However, recent *in vivo* data using receptor-mediated signal measurement approaches reveal that the strength of M4R synaptic transmission on direct pathway medium spiny neurons (MSN) is diminished in DA-depleted mice. Restoration of M4R signaling partially alleviated motor deficits and LID, indicating that reduced M4R function may differentially influence PD and LID pathophysiology and could represent a promising therapeutic target.^146^

Beyond ACh receptors, VAChTs are also under investigation in PD. In a retrospective cross-sectional study investigating the correlation between visual contrast sensitivity and regional cerebral cholinergic vesicular transporters, it was found that cholinergic deficits in the brain are associated with impaired contrast sensitivity in PD. Moreover, lower Rabin contrast sensitivity scores were linked to poorer overall scores on the PD Cognitive Rating Scale, suggesting that reduced cognitive performance related to contrast sensitivity may partly reflect underlying cholinergic system weaknesses.^147^

Genetic mutations such as *GBA1* and *LRRK2*, which are associated with different clinical phenotypes in PD, are also related to cholinergic system involvement. Research examining the association between these mutations and cholinergic system integrity found that both were linked to increased basal forebrain volume in asymptomatic stages. However, this volumetric increase persisted only in symptomatic *LRRK2* carriers, where it was associated with slower cognitive decline.^148^ Additionally, a clinical study of cognitive decline in PD demonstrated that atrophy of the cholinergic basal forebrain predicts cognitive deterioration. Volumetric assessment of the nucleus basalis of Meynert—a cholinergic neuronal structure in the forebrain—may offer early indicators of cognitive impairment in PD. The differentiated associations between basal forebrain status and domain-specific cognitive decline may have implications for understanding the neural basis of PD-related cognitive heterogeneity.^149^

The loss of nigral dopaminergic neurons in PD creates an imbalance between cholinergic interneurons and dopaminergic inputs in the striatum.^150^ A clinical neuroimaging study using Dual-Tracer PET and dopaminergic PET-supported correlative tractography found evidence of striatal ACh-DA imbalance in early-stage PD. Furthermore, increased ACh-DA imbalance in the more affected hemisphere correlated with higher bradykinesia scores. Therapeutic strategies aimed at restoring ACh-DA balance may thus represent a crucial step in PD treatment.^151^ The role of glutamate in the motor symptoms of PD through interactions with the cholinergic system has also been studied. In a 6-OHDA mouse model, loss of nigral stimulation of cholinergic interneurons, which regulate striatal DA and ACh transmission along with dopaminergic inputs, was shown to downregulate mGluR1 receptors on these interneurons in dorsolateral striatum, abolishing DA excitatory influence. Restoration of mGluR1 signaling in cholinergic neurons was sufficient to recover circuit function and alleviate motor deficits in early-stage PD mice.^152^

In a phase 2 randomized clinical trial, the efficacy of TAK-071, a positive allosteric modulator of the M1R, was evaluated in PD patients with fall risk and cognitive impairment. TAK-071 was well tolerated in this patient population, and while it did not improve the primary outcome of gait variability, it did enhance cognitive performance compared to placebo. Further studies with larger cohorts and longer durations are needed to fully evaluate the safety and efficacy of TAK-071 as a therapeutic agent.^153^

In a PD rat model, treatment with the cholinesterase and butyrylcholinesterase inhibitor rivastigmine, either alone or in combination with the selective 5-HT6 antagonist idalopirdine, was shown to reduce the fall tendencies. Rivastigmine alone showed strong tendencies to reduce slips and falls, while the combination therapy was more effective than rivastigmine alone in reducing stop-related falls. Idalopirdine alone was found to be ineffective. These findings suggest that combination therapy with idalopirdine and a cholinesterase inhibitor may improve complex motor control and reduce fall risk in patients with movement disorders. Targeting cholinergic system receptors and transporter proteins may be valuable for developing novel therapeutic approaches (Figure 3A).^154^

**Figure 3:**
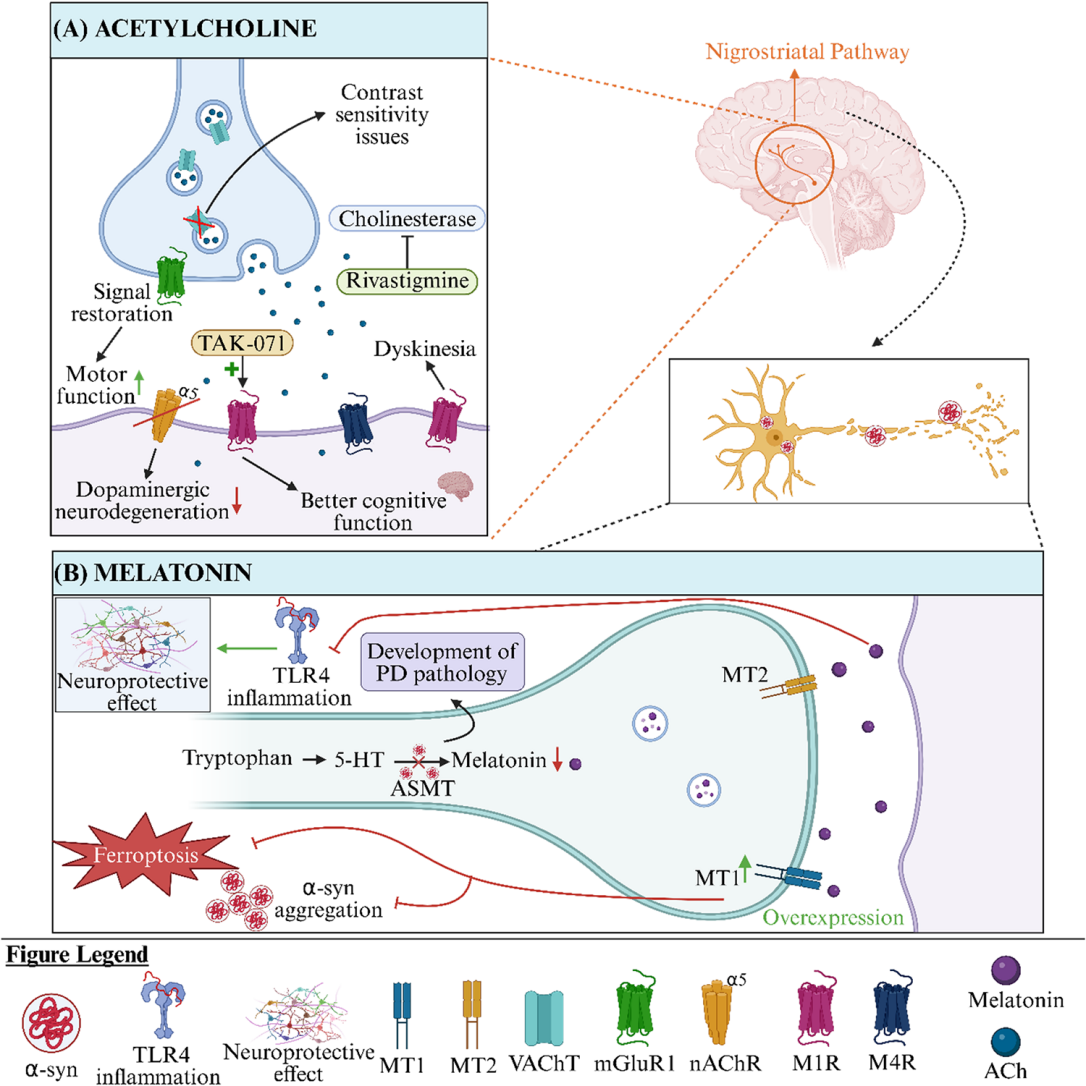
(A) The role of ACh in PD. TAK-071, a positive allosteric M1R modulator, improved cognitive function in PD patients compared to placebo.^153^ Rivastigmine alone showed strong tendencies to reduce slips and falls, while the combination therapy with idalopirdine was more effective than rivastigmine alone in reducing stop-related falls.^154^ A study examining the relationship between regional cerebral VAChT levels and visual contrast sensitivity revealed that cholinergic deficits are associated with reduced contrast sensitivity in PD patients.^147^ In an in vivo study, the absence of α5 nAChR in hemiparkinsonian mice was associated with reduced dopaminergic neurodegeneration and motor impairments.^144^ In another study, the loss of nigral stimulation was shown to lead to downregulation of mGluR1 receptors in cholinergic interneurons in the dorsolateral striatum, abolishing the excitatory effect of dopamine. Restoration of mGluR1 signaling in cholinergic interneurons was sufficient to improve circuit function and reduce motor impairments in early-stage PD model mice.^152^ (B) The role of melatonin in PD. α-synuclein was found to increase binding to ASMT, the enzyme catalyzing the final step of melatonin biosynthesis via interaction with light chain 3B, leading to impaired ASMT activity and reduced melatonin synthesis.^166^ Melatonin has been shown to reduce α-syn aggregation and TLR4-mediated inflammatory response.^163^ Overexpression of MT1 inhibits α-synuclein aggregation and ferroptosis, highlighting a novel mechanism involving melatonin receptors and iron metabolism in PD pathogenesis.^162^ *Ach: acetylcholine*, *ASMT: acetylserotonin-O-methyltransferase*, *M1R: muscarinic acetylcholine receptor 1*, *M4R: muscarinic acetylcholine receptor 4*, *mGluR1: metabotropic glutamate receptor 1*, *MT: melatonin*, *MT1: Melatonin receptor 1*, *MT2: Melatonin receptor 2*, *nAChR: nicotinic acetylcholine receptor*, *TLR4: Toll Like Receptor 4*, *α-syn: α-synuclein*.

In conclusion, in addition to the dopaminergic system, the cholinergic system is another neurotransmitter pathway significantly involved in the pathophysiology of PD. Dysfunction of its receptors and transporters can affect various components of the disease. Moreover, genetic mutations affecting this system, disturbances in cholinergic neurons, and interactions with other neurotransmitter systems are being investigated as therapeutic targets for PD. A deeper understanding of this system may pave the way for the development of new treatments (Table 7).

**Table 7:** Summary of the Findings Reporting the Role of Acetylcholine in PD^a^

s.n.	study type	PD model	drug and dose	observation	remarks	references
1	*In vitro*	6-OHDA induced α5-knockout C57BL/6J mice and wild-type littermates		The absence of α5 nAChR in hemiparkinsonian mice was associated with reduced dopaminergic neurodegeneration and motor dysfunction	nAChRs containing the α5 subunit may represent novel therapeutic targets for PD	^144^
2	*In vivo*	6-OHDA induced Sprague–Dawley rats treated with 6 mg/kg L-DOPA (plus 12 mg/kg benserazide, s.c.)	Telenzepine (100 nM), PD-102807 (3 μM), Tropicamide (100 nM), VU0152100 (100 μM), AFDX-116 (200 nM)	Striatal and nigral M1R and M4Rs have been shown to modulate LID and GABAergic activation of the striato-nigral pathway	Striatal M1Rs facilitate *in vivo* dyskinesia and activation of the striato-nigral pathway, whereas striatal M4Rs may either facilitate or inhibit dyskinesia depending on their localization	^145^
3	*In vivo*	6-OHDA induced DAT-IRES-Cre heterozygote mice, ChAT-IRES-Cre heterozygote mice, VGLUT2 conditional knockdown, CaMKII-Cre heterozygote mice and wild-type C57BL/6J mice		Loss of nigral stimulation of cholinergic interneurons was shown to downregulate mGluR1 receptors on these interneurons in dorsolateral striatum, abolishing DA excitatory influence	Restoration of mGluR1 signaling in cholinergic neurons is sufficient to recover circuit function and alleviate motor deficits in early-stage PD mice	^152^
4	*In vivo*	6-OHDA induced wild-type C57BL/6J mice, Adora2a-Cre heterozygote mice, Drd1-Cre heterozygote mice, RGS4 cKO in D1-MSNs and littermate controls, MitoPark and littermate control mice treated with l-DOPA (2 mg/kg, i.p.) plus benserazide hydrochloride (12 mg/kg)		The strength of M4 receptor synaptic transmission on direct pathway MSNs is diminished in DA-depleted mice	Restoration of M4 signaling partially alleviated motor deficits and LID indicating that reduced M4 function may differentially influence PD and LID pathophysiology and could represent a promising therapeutic target	^146^
5	*In vivo*	6-OHDA induced Sprague–Dawley rats	Rivastigmine (0.1, 0.3, or 1.0 mg/kg), Idalopirdine (10.0 mg/kg)	Rivastigmine alone showed strong tendencies to reduce slips and falls, while the combination therapy was more effective than rivastigmine alone in reducing stop-related falls whereas idalopirdine alone was found to be ineffective	Combination therapy with idalopirdine and a cholinesterase inhibitor may improve complex motor control and reduce fall risk in patients with movement disorders	^154^
6	*Observational Human study*	PD patients (*n* = 91)		Cholinergic deficits in the brain are associated with impaired contrast sensitivity in PD, lower Rabin contrast sensitivity scores are linked to poorer overall scores on the PD Cognitive Rating Scale	Reduced cognitive performance related to contrast sensitivity may partly reflect underlying cholinergic system weaknesses	^147^
7	*Observational Human study*	PD patients with different mutations (*n* = 490), idiopathic PD patients (*n* = 492), HC (*n* = 180)		Both *GBA1* and *LRRK2* mutations are linked to increased basal forebrain volume in asymptomatic stages	The volumetric increase persisted only in symptomatic *LRRK2* carriers, where it was associated with slower cognitive decline	^148^
8	*Human study*	PD patients (*n* = 168), HC (*n* = 76)		Atrophy of the cholinergic basal forebrain predicts cognitive deterioration and volumetric assessment of the nucleus basalis of Meynert may offer early indicators of cognitive impairment in PD	The differentiated associations between basal forebrain status and domain-specific cognitive decline may have implications for understanding the neural basis of PD-related cognitive heterogeneity	^149^
9	*Observational Human study*	PD patients (*n* = 45), HC (*n* = 15)		Striatal ACh-DA imbalance is seen in early-stage PD and increased ACh-DA imbalance in the more affected hemisphere is correlated with higher bradykinesia scores	Therapeutic strategies aimed at restoring ACh-DA balance may thus represent a crucial step in PD treatment	^151^
10	*Interventional (Randomized) Human study*	PD patients (*n* = 54)	Once-daily oral TAK-071 (5.0–7.5 mg)	TAK-071 was well tolerated in PD patients with fall risk and cognitive impairment and, while it did not improve the primary outcome of gait variability, it did enhance cognitive performance compared to placebo	Further studies with larger cohorts and longer durations are needed to fully evaluate the safety and efficacy of TAK-071 as a therapeutic agent	^153^

a 6-OHDA: 6-hydroxydopamine, ACh: acetylcholine, DA: dopamine, GABA: γ-aminobutyric acid, LID: L-DOPA-induced dyskinesia, M1R: muscarinic acetylcholine receptor 1, M4R: muscarinic acetylcholine receptor 4, MSNs: medium spiny neurons, mGluR: metabotropic glutamate receptor, nAChR: nicotinic acetylcholine receptor, PD: Parkinson’s disease.

## 9 Melatonin

Melatonin is defined as an indoleamine-structured neurotransmitter and a hormone synthesized primarily by the pineal gland and other tissues. The neurotransmitter contributes to neuroprotection, regulation of circadian rhythms, and synchronization of the sleep-wake cycle.^155^ Melatonin is synthesized mainly in the pinealocytes of the pineal gland through the methylation of N-acetylserotonin, which is formed by the acetylation of 5-HT, a derivative of tryptophan, by aralkylamine N-acetyltransferase, followed by methylation via acetylserotonin-O-methyltransferase (ASMT).^156^ The production of melatonin is regulated by the transmission of light information from the retina to the pineal gland through the suprachiasmatic nucleus of the hypothalamus. Melatonin levels increase during the night and decrease during the day.^157^

Melatonin exerts its physiological effects through melatonin receptor 1 (MT1) and melatonin receptor 1 (MT2) receptors, which are high-affinity G protein-coupled receptors.^158,159^ Additionally, as a free radical scavenger, melatonin enhances mitochondrial homeostasis and supports the survival of dopaminergic neurons. Dysregulation in the melatonergic system can lead to pathologies such as increased α-syn, causing damage to the dopaminergic system.^160^ In a 6-OHDA-induced PD model, the role of MT2 receptors in modulating depressive-like behavior and olfaction has been explored. It was shown that MT1 and MT2 receptors are coexpressed in dopaminergic neurons within the glomerular layer of the olfactory bulb and are functionally linked to Gi proteins. A translational approach delivering melatonin intranasally, directly to the olfactory bulb, may aid in the treatment of depression in PD patients.^161^

In a study using C57BL/6JGpt-Mtnr1aem3Cd3121/Gpt mice, the role of MT1 receptors in PD was investigated. The study revealed that MT1 overexpression inhibited α-syn aggregation and ferroptosis, a form of iron-dependent cell death, highlighting a novel mechanism involving melatonin receptors and iron metabolism in PD pathogenesis. A reduction in MT1 expression may contribute to PD pathogenesis.^162^ In an MPTP-induced PD mouse model, the effect of melatonin on α-syn aggregation was examined. Melatonin helped to reduce α-syn aggregation and toll like receptor 4 (TLR4)-mediated inflammatory responses. Although there is no direct evidence for the involvement of the TLR4 pathway in PD, melatonin may exert neuroprotective effects.^163^

In a 6-OHDA-induced PD mouse model, the effects of melatonin on the motor activity and oxidative stress parameters were investigated. The study showed that mice treated with melatonin before 6-OHDA injection exhibited less dopaminergic neuronal death compared with those treated after the injection. However, melatonin treatment did not significantly affect the locomotor activity. These findings suggest that while melatonin has potential as a neuroprotective agent in PD, it may not be sufficient alone to improve motor symptoms.^164^ Similarly, the effects of melatonin administered before and after MPTP induction were studied in zebrafish embryos. Melatonin exhibited neuroprotective properties when given before MPTP induction and helped to restore motor function when administered afterward. In addition to preventing neuronal degeneration, these findings support the therapeutic potential of melatonin in PD treatment.^165^

Since RBD-like behaviors are among the nonmotor symptoms of PD, the relationship between α-syn and melatonin has been investigated. α-syn was found to increase binding to ASMT, the enzyme catalyzing the final step of melatonin biosynthesis via interaction with light chain 3B, leading to impaired ASMT activity and reduced melatonin synthesis. Disruption of melatonin synthesis by α-synuclein may help elucidate the molecular mechanisms of RBD-like behaviors in α-syn-based transgenic mice and could provide a novel therapeutic target for the disease.^166^

*In vivo* and *in vitro* studies investigated the anti-inflammatory properties of melatonin in BV2 microglial cell lines and PD mouse models induced by α-syn fibrils. The study demonstrated that melatonin inhibited NLRP3 inflammasome activation induced by α-syn fibrils via the toll like receptor 2 (TLR2) pathway in microglia. By reducing neuroinflammation, melatonin was shown to protect against dopaminergic neuron loss, suggesting its potential role in anti-inflammatory treatment strategies for PD.^167^ Another *in vivo* and *in vitro* study examined the delaying effects of melatonin-loaded nanoparticles in PD. The study revealed a novel mechanism by which melatonin-loaded nanoparticles mediate PTEN degradation and stimulate mitophagy through the regulation of BMI-1, a gene involved in the maintenance and differentiation of neurogenic tissues. The molecular and cellular dynamics influenced by nanomelatonin to regulate mitophagy may serve as a potential therapeutic avenue in PD.^168^ The neuroprotective effects of melatonin were also studied in a homocysteine (Hcy)-based PD model. It was shown that melatonin dose-dependently reversed DA depletion in the striatum, tyrosine hydroxylase-positive neuronal loss in the substantia nigra, and oxidative stress in the substantia nigra. These findings suggest that the antioxidant and radical scavenging properties of melatonin contribute to its neuroprotective effects, supporting its therapeutic potential in PD.^169^ In a study examining melatonin levels in PD patients, a 6-OHDA-induced rat model, and HCs, the potential of melatonin as an alternative index for PD severity was investigated. The results showed significantly higher melatonin levels in both PD patients and rats compared to HCs. Additionally, serum melatonin levels were found to correlate with PD severity according to the H&Y scale. Melatonin may thus be useful in monitoring the prognosis of PD patients (Figure 3B).^170^

In conclusion, melatonin emerges as a promising therapeutic agent in PD due to its neuroprotective, antioxidant, and anti-inflammatory properties. Current research indicates that melatonin may protect dopaminergic neurons, prevent α-syn aggregation, and reduce oxidative stress. These findings provide new hope for the clinical application of melatonin in PD treatment (Table 8).

**Table 8:** Summary of Findings Reporting the Role of Melatonin in PD^a^

s. n.	study type	PD model	drug and dose	observation	remarks	references
1	*In vivo*	6-OHDA	Melatonin (MLT, 1 μg/μL)	It was shown that MT1 and MT2 receptors are coexpressed in dopaminergic neurons within the glomerular layer of the olfactory bulb and are functionally linked to Gi proteins.	A translational approach delivering melatonin intranasally, directly to the olfactory bulb, may aid in the treatment of depression in PD patients.	^161^
		Wistar rats	LUZ (5 μg/μL), a MT2 receptor antagonist			
			4-P-PDOT (5 μg/μL), a selective MT2 receptor drug			
2	*In vivo*	C57BL/6JGpt-Mtnr1a^em3Cd3121^/Gpt mice		The study revealed that MT1 overexpression inhibited α-synuclein aggregation and ferroptosis, a form of iron-dependent cell death, highlighting a novel mechanism involving melatonin receptors and iron metabolism in PD pathogenesis.	A reduction in MT1 expression may contribute to PD pathogenesis.	^162^
3	*In vivo*	MPTP-induced C57BL/6 mice	Melatonin (20 mg/kg/day)	Melatonin has been shown to reduce α-syn aggregation and TLR4-mediated inflammatory response.	Although there is no direct evidence for the involvement of the TLR4 pathway in PD, melatonin may exert neuroprotective effects.	^163^
4	*In vivo*	6-OHDA induced rats	Melatonin (10 mg/kg/gün)	The study showed that mice treated with melatonin before 6-OHDA injection exhibited less dopaminergic neuronal death compared to those treated after the injection. However, melatonin treatment did not significantly affect locomotor activity.	These findings suggest that while melatonin has potential as a neuroprotective agent in PD, it may not be sufficient alone to improve motor symptoms.	^164^
5	*In vivo*	MPTP-induced zebrafish	Melatonin (0.2 and 1 μm)	Melatonin exhibited neuroprotective properties when given before MPTP induction and helped restore motor function when administered afterward.	In addition to preventing neuronal degeneration, these findings support the therapeutic potential of melatonin in PD treatment.	^165^
6	*In vivo*	B6;C3-Tg 83Vle/J mice	Melatonin (10 mg/kg/day)	α-synuclein was found to increase binding to ASMT, the enzyme catalyzing the final step of melatonin biosynthesis via interaction with light chain 3B, leading to impaired ASMT activity and reduced melatonin synthesis.	Disruption of melatonin synthesis by α-synuclein may help elucidate the molecular mechanisms of RBD-like behaviors in α-synuclein-based transgenic mice and could provide a novel therapeutic target for the disease.	^166^
7	*In vivo*	Hcy rat model	Melatonin (10, 20, and 30 mg/kg, i.p.)	It was shown that melatonin dose-dependently reversed dopamine depletion in the striatum, tyrosine hydroxylase-positive neuronal loss in the substantia nigra, and oxidative stress in the substantia nigra	These findings suggest that the antioxidant and radical scavenging properties of melatonin contribute to its neuroprotective effects, supporting its therapeutic potential in PD.	^169^
8	*In vitro and ın vivo*	α-syn fibrils induced BV2 cells and Mouse model	Melatonin	The study demonstrated that melatonin inhibited NLRP3 inflammasome activation induced by α-synuclein fibrils via the TLR2 pathway in microglia.	By reducing neuroinflammation, melatonin was shown to protect against dopaminergic neuron loss, suggesting its potential role in anti-inflammatory treatment strategies for PD.	^167^
9	*In vivo and observational human study*	6-OHDA Sprague–Dawley rats		The results showed significantly higher melatonin levels in both PD patients and rats compared to HCs.	Additionally, serum melatonin levels were found to correlate with PD severity according to the H&Y scale. Melatonin may thus be useful in monitoring the prognosis of PD patients.	^170^
		PD patient (*n* = 56)				
		HC (*n* = 22)				

a ASMT: acetylserotonin-O-methyltransferase, Hcy: homocystein, MT: melatonin, TLR4: Toll-like receptor 4, PD: Parkinson’s disease, RBD: rapid eye movement sleep behavior disorder, α-syn: α-synuclein.

## 10 Nitric Oxide

Nitric oxide (NO) is a small, lipophilic molecule composed of one nitrogen and one oxygen atom, playing a crucial role in intercellular signal transduction.^171^ In the human body, NO is synthesized from the amino acid l-arginine by members of the NO synthase (NOS) enzyme family.^172^ The NOS family comprises three isoforms with distinct physiological roles: neuronal NOS (nNOS), endothelial NOS (eNOS), and inducible NOS (iNOS). nNOS is primarily found in the CNS and PNS and is involved in neurotransmission. eNOS, located in the vascular endothelium, regulates vascular tone, while iNOS is induced in immune cells and helps in host defense mechanisms.^173^

The activation of guanylate cyclase by NO leads to increased production of cyclic guanosine monophosphate (cGMP), which modulates various physiological processes such as vasodilation, neurotransmission, and immune response.^174^ In addition to its physiological roles, excessive NO production can be detrimental. Under pro-oxidant conditions, NO can react with ROSs to form reactive nitrogen species (RNS), which contribute to cellular damage.^175^ This process, known as nitrosative stress, is implicated in the pathogenesis of neurodegenerative diseases.^176^

nNOS, the primary source of NO in neurons, has been shown to be upregulated in post-mortem analyses and animal models of PD.^172^ A study investigating the association between nNOS polymorphisms and susceptibility to oxidative stress in PD found that while the TT genotype in exon 29 of the nNOS gene was significantly associated with the disease, overall, nNOS polymorphisms contributed minimally to PD risk. Furthermore, increased lipid peroxidation and genotype-dependent changes in nitrite levels were observed in PD patients.^177^ These findings suggest that NO production and oxidative stress may play roles in PD pathogenesis. An observational study exploring the relationship between nNOS gene polymorphisms and LID in PD patients examined the rs2682826 single nucleotide polymorphism (SNP) in the *NOS1* gene, which encodes nNOS. The study concluded that this SNP did not significantly influence LID susceptibility or severity.^178^ More comprehensive studies investigating a broader spectrum of nitric oxide synthase 1 (NOS1) variants are necessary to fully elucidate the gene’s role in LID.

The involvement of the iNOS isoform in cardiovascular and autonomic changes was evaluated in male rats with induced parkinsonism. iNOS inhibition did not provide neuroprotection against striatal dopaminergic loss, yet iNOS appeared to contribute to vascular hypo-reactivity in Parkinsonian animals.^179^ Cardiovascular dysfunction in parkinsonism may not be limited to the CNS but could also involve peripheral factors including endothelial iNOS encoded by the *NOS2* gene. In a transgenic synucleinopathy mouse model, genetic deletion of nitric oxide synthase 2 (NOS2) ameliorated α-syn pathology and associated neuroinflammatory responses, suggesting its potential as a therapeutic target for modulating PD pathology.^180^

In a 6-OHDA induced PD rat model, the effects of the natural polyphenol mangiferin, known for its potent antioxidant and anti-inflammatory properties, were studied both alone and in combination with the nNOS inhibitor 7-nitroindazole (7-NI). The combination treatment significantly improved locomotor parameters in the rats. Notably, 7-NI suppressed tumor necrosis factor-α (TNF-α) and interleukin-6 (IL-6) mRNA expression via nNOS inhibition, enhancing the anti-inflammatory and antiparkinsonian effects of mangiferin and contributing to oxidative stress reduction.^181^ The level of tyrosine hydroxylase expression induced by the combination therapy was comparable to that of levodopa, suggesting an alternative approach to enhance DA preservation and bioavailability. Another study in a 6-OHDA induced PD rat model investigated the effects of long-term nNOS inhibition on the LID development and expression. An increased number of nNOS-expressing interneurons in the lateral striatum was associated with LID. Long-term administration of 7-NI reduced the number of these interneurons, indicating that NOS inhibitors may offer a novel therapeutic strategy for alleviating LID.^182^ Research into the mechanisms of neuronal cell death in PD has identified necroptosis as a predominant pathway in disease progression. NO has been shown to trigger necroptosis, also referred to as programmed necrosis, suggesting that targeting NO-mediated necroptotic pathways may offer promising therapeutic opportunities for neurodegenerative diseases such as PD.^183^

An *in vivo* mouse study utilizing an activated fluorescent probe to monitor NO fluctuations in PD demonstrated that NO levels progressively increased in the brains of PD mice as the disease worsened, confirming a close association between NO concentration and PD progression.^184^ Another Golgi-targeted fluorescent probe (Gol-NO) developed for *in vivo* and *in vitro* imaging of NO in PD models also revealed increased NO concentrations in cells and zebrafish PD models.^185^ Furthermore, a photoacoustic probe developed by Jiang et al. successfully crossed the BBB and showed NO to be predominantly distributed in the cerebral cortex of MPTP-induced PD rats.^186^ The development of such tools to measure NO may further advance our understanding of PD diagnosis and treatment (Figure 4).

**Figure 4:**
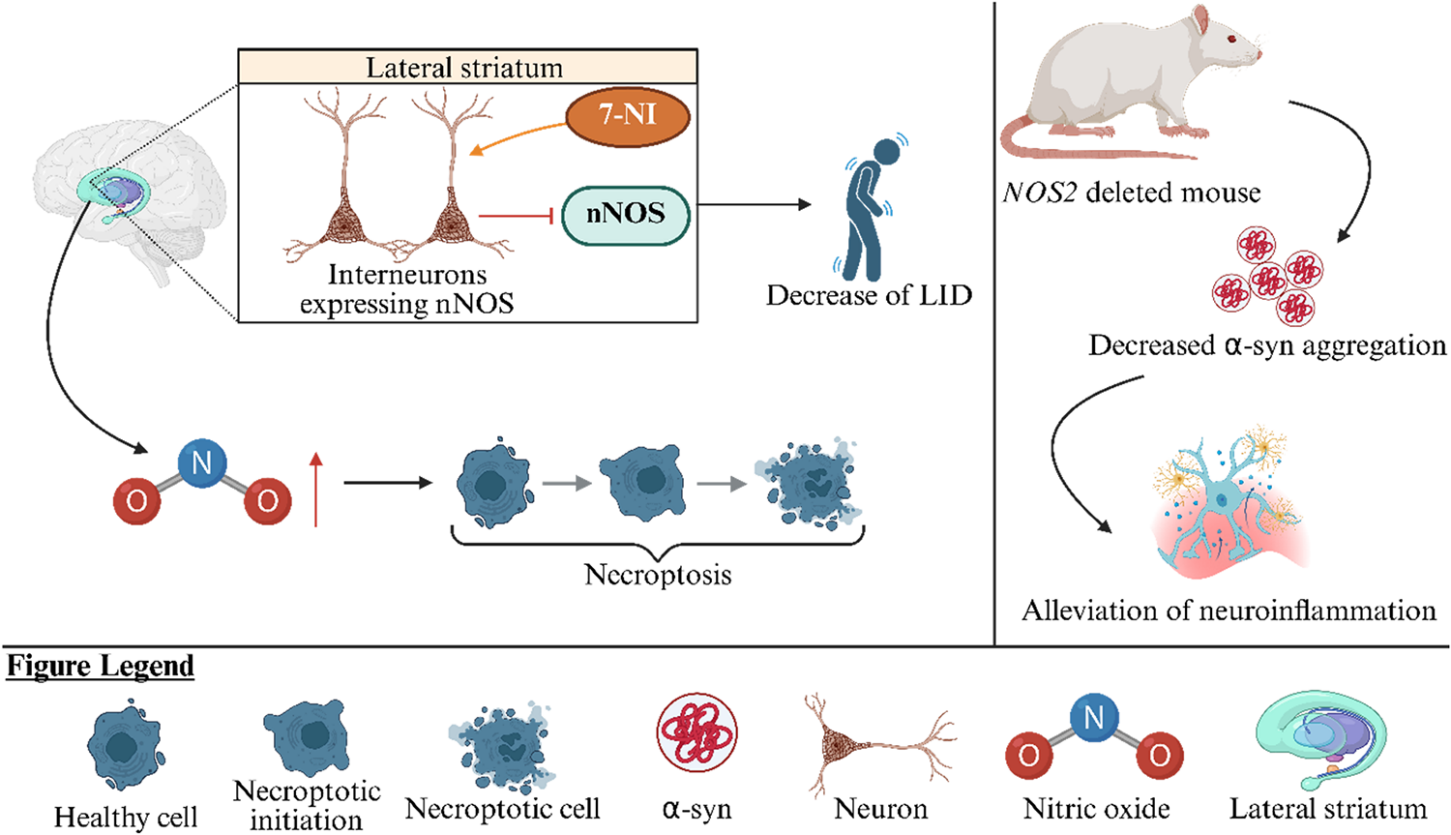
The role of NO in PD. An increased number of nNOS-expressing interneurons in the lateral striatum was associated with LID. Long-term administration of 7-NI reduced the number of these interneurons, indicating that NOS inhibitors may offer a novel therapeutic strategy for alleviating LID.^182^ NO has been shown to trigger necroptosis, also referred to as programmed necrosis, suggesting that targeting NO-mediated necroptotic pathways may offer promising therapeutic opportunities for neurodegenerative diseases such as PD.^183^ In a transgenic synucleinopathy mouse model, genetic deletion of NOS2 ameliorated α-synuclein pathology and associated neuroinflammatory responses.^180^ *7-NI: 7-nitro-indazole*, *LID: L-DOPA-induced dyskinesia*, *nNOS: neuronal NOS*, *NO: Nitric oxide*, *NOS: NO synthase*, *α-syn: α-synuclein*.

In conclusion, NO contributes to oxidative stress, neuroinflammation, and neurodegeneration in PD pathogenesis. Elevated NO levels are associated with the overexpression of nNOS and iNOS, and nNOS inhibition has been shown to exert neuroprotective effects and alleviate LID. Targeting NO-mediated signaling pathways presents a promising therapeutic approach for slowing the progression of PD and managing its symptoms (Table 9).

**Table 9:** Summary of the Findings Reporting the Role of Nitric Oxide in PD^a^

s. n.	study type	PD model	drug and dose	observation	remarks	refs
1	*In vivo*	6-OHDA induced rats	iNOS inhibitor, S-metilizotiyoüre (SMT; 10 mg/kg; i.p.)	iNOS inhibition did not provide neuroprotection against striatal dopaminergic loss, yet iNOS appeared to contribute to vascular hypo-reactivity in Parkinsonian animals.	Cardiovascular dysfunction in parkinsonism may not be limited to the CNS but could also involve peripheral factors, including endothelial iNOS encoded by the *NOS2* gene.	^179^
2	*In vivo*	Syn^A53T^ Transgenic mice		In a transgenic synucleinopathy mouse model, genetic deletion of NOS2 ameliorated α-synuclein pathology and associated neuroinflammatory responses.	The study’s findings may indicate a therapeutic target for modulating PD pathology in the brain.	^180^
3	*In vivo*	6-OHDA lesioned Wistar rats	Mangiferin (45 μg)	The effects of the natural polyphenol mangiferin, known for its potent antioxidant and anti-inflammatory properties, were studied both alone and in combination with the nNOS inhibitor 7-NI. The combination treatment significantly improved locomotor parameters in the rats.	Notably, 7-NI suppressed TNF-α and IL-6 mRNA expression via nNOS inhibition, enhancing the anti-inflammatory and antiparkinsonian effects of mangiferin and contributing to oxidative stress reduction.	^181^
			7-NI (10 mg/kg)			
4	*In vivo*	6-OHDA induced Wistar rats	7-NI (30 mg/kg)	An increased number of nNOS-expressing interneurons in the lateral striatum was associated with LID. Long-term administration of 7-NI reduced the number of these interneurons, indicating that NOS inhibitors may offer a novel therapeutic strategy for alleviating LID.	The development of NOS-inhibiting compounds may be a new therapeutic avenue for alleviating LID.	^182^
5	*In vivo*	Mouse	PO-NH (100 μmol/L 150 μL)	An *in vivo* mouse study utilizing an activated fluorescent probe to monitor NO fluctuations in PD demonstrated that NO levels progressively increased in the brains of PD mice as the disease worsened.	The study results confirmed that the change in NO concentration is closely related to the development of PD.	^184^
6	*In vitro and in vivo*	Rotenone-induced cell and zebrafish PD models	Gol-NO (5 μM, 0.5 h)	Another Gol-NO developed for *in vivo* and *in vitro* imaging of NO in PD models also revealed increased NO concentrations in cells and zebrafish PD models.	As a result of the study, it was shown that NO concentration increased in the PD group with Gol-NO in cell and zebrafish PD models.	^185^
7	*In vitro and in vivo*	C57BL/6 rat and SH-SY5Y cell		NO has been shown to trigger necroptosis, also referred to as programmed necrosis, suggesting that targeting NO-mediated necroptotic pathways may offer promising therapeutic opportunities for neurodegenerative diseases such as PD.	Targeting the NO-mediated necroptosis pathway in treatment planning may offer promising avenues to combat neurodegenerative diseases such as PD.	^183^
8	*Observational Human Study*	PD patient (*n* = 89)		A study investigating the association between nNOS polymorphisms and susceptibility to oxidative stress in PD found that while the TT genotype in exon 29 of the nNOS gene was significantly associated with the disease, overall, nNOS polymorphisms contributed minimally to PD risk.	Furthermore, increased lipid peroxidation and genotype-dependent changes in nitrite levels were observed in PD patients.	^177^
		Case-control (*n* = 332)				
9	*Observational Human Study*	PD with dyskinesia (*n* = 91)		An observational study exploring the relationship between nNOS gene polymorphisms and LID in PD patients examined the rs2682826 single nucleotide polymorphism in the *NOS1* gene, which encodes nNOS. The study concluded that this SNP did not significantly influence LID susceptibility or severity.	More comprehensive studies investigating a broader spectrum of NOS1 variants are necessary to fully elucidate the gene’s role in LID.	^178^
		PD without dyskinesia (*n* = 95)				

a 7-NI: 7-nitro-indazole, BBB: blood–brain barrier, CNS: central nervous system, DA: dopamine, Gol-NO: golgi-targeted fluorescent probe, iNOS: inducible NOS, IL-6: interleukin-6, LID: L-DOPA-induced dyskinesia, nNOS: neuronal NOS, NO: Nitric oxide, NOS: NO synthase, PD: Parkinson’s Disease, TNF-α: tumor necrosis factor α, α-syn: α-synuclein.

## 11 Adenosine

Adenosine, a nucleoside primarily formed through the extracellular hydrolysis of adenine nucleotides, exerts significant neuromodulatory effects in the CNS via four G protein-coupled receptor subtypes: A_1_ receptor (A_1_R), A_2_A receptor (A_2_AR), A_2_B receptor (A_2_BR), and A_3_ receptor (A_3_R).^187,188^ Among these, the A_2_A receptor (A_2_AR) is notably enriched in the striatum, particularly within the caudate–putamen regions densely innervated by dopaminergic neurons.^189^ A_2_ARs are predominantly expressed in the indirect pathway MSNs, where they modulate motor control by antagonizing D_2_R signaling, thereby contributing to the motor symptoms observed in PD.^190^

The therapeutic potential of A_2_AR antagonists in PD has been substantiated by animal studies evaluating istradefylline, a selective A_2_AR antagonist. Istradefylline has demonstrated efficacy in reducing ″OFF″ time and improving motor function in PD patients when used adjunctively with levodopa, without exacerbating dyskinesia.^191^

Genetic studies have identified mutations in adenosine receptors that may influence PD pathophysiology. A notable example is the G279S mutation in A_1_R, which results in a receptor variant with altered structural activity. This mutation may lead to prolonged activation of signaling pathways that support neurodegeneration, potentially increasing susceptibility to PD.^192^ Interactions between adenosine receptors and other G protein-coupled receptors also play a role in PD. GPR37, an orphan receptor associated with PD neuropathology, has been shown to interact with A_2_ARs in the striatum. Deletion of GPR37 enhances A_2_AR cell surface expression and function, leading to increased cyclic adenosine monophosphate (cAMP) accumulation in response to A_2_AR agonists. This suggests that GPR37 negatively regulates A_2_AR signaling, and its absence may exacerbate A_2_AR-mediated effects, potentially influencing PD progression.^193^ Furthermore, adenosine and DA signaling converge on the regulation of protein kinase A (PKA) activity in striatal neurons. Adenosine, via A_2_AR activation, stimulates PKA in D_2_R-expressing MSNs, while DA, through D_2_R activation, inhibits PKA. The balance between these opposing signals is crucial for normal motor function, and disruptions may contribute to the motor deficits characteristic of PD.^194^

In summary, adenosine, particularly through A_2_ARs, plays a significant role in modulating motor circuits affected in PD. Therapeutic strategies targeting adenosine receptors, such as the use of A_2_AR antagonists like istradefylline, offer promising avenues for alleviating motor symptoms in PD. Additionally, understanding the genetic and molecular interactions involving adenosine receptors may provide further insights into PD pathogenesis and treatment (Table 10).

**Table 10:** Summary of Findings Reporting the Role of Adenosine in PD^a^

s.n.	study type	PD model	drug and dose	observation	remarks	references
1	*In vitro*	HEK293 cells		A notable example is the G279S mutation in the A_1_R, which results in a receptor variant with altered structural activity	This mutation may lead to prolonged activation of signaling pathways that support neurodegeneration, potentially increasing susceptibility to PD.	^192^
2	*In vitro*, *In vivo*	C57BL/6J mice, Primary striatal neuron cell culture		Deletion of GPR37 enhances A_2_AR cell surface expression and function, leading to increased cAMP accumulation in response to A_2_AR agonists.	This suggests that GPR37 negatively regulates A_2_AR signaling, and its absence may exacerbate A_2_AR-mediated effects, potentially influencing PD progression.	^193^
3	*In vivo*	MPTP-induced marmosets	Ropinirole (0.025–0.075 mg/kg p.o.), Pergolide (0.01 mg/kg p.o.)	The therapeutic potential of A_2_AR antagonists in PD has been substantiated by animal studies evaluating istradefylline, a selective A_2_AR antagonist.	Istradefylline has demonstrated efficacy in reducing “OFF” time and improving motor function in PD patients when used adjunctively with levodopa, without exacerbating dyskinesia.	^191^
			l-DOPA (2.5 mg/kg p.o.), Istradefylline (10 mg/kg p.o.),			
4	*In vivo*	*Drd1a-cre*, *Adora2a-cre*, *DAT-IRES-cre* transgenic mice, Wild-type C57BL/6 mice	Rp-8-Br-cAMPS	Adenosine, via A_2_AR activation, stimulates PKA in D_2_R-expressing MSNs, while DA, through D_2_R activation, inhibits PKA.	The balance between these opposing signals is crucial for normal motor function, and disruptions may contribute to the motor deficits characteristic of PD.	^194^
			H89 istradefylline SCH58261			

a A_1_R: A_1_ receptor, A_2_AR: A_2_A receptor, cAMP: cyclic adenosine monophosphate, D2R: D2 receptor, DA: dopamine, L-DOPA: L-3,4-dihydroxyphenylalanine, MPTP: 1-methyl-4-phenyl-1,2,3,6-tetrahydropyridine, MSNs: medium spiny neurons, PKA: protein kinase A, PD: Parkinson’s disease.

## 12 Current Therapeutic Strategies and Potential Limitations Associated with Neurotransmitter-Based Treatments in PD

L-DOPA remains the gold standard for the treatment of motor symptoms in PD.^195^ Co-administration of L-DOPA with peripheral dopa decarboxylase (DDC) inhibitors such as carbidopa or benserazide increases the amount of L-DOPA reaching the brain.^196^ In addition, catechol-O-methyltransferase (COMT) inhibitors such as entacapone and opicapone prolong the half-life of L-DOPA; however, tolcapone is only used to a limited extent due to the risk of hepatotoxicity.^197^ In addition, DA agonists are used as monotherapy or in combination with L-DOPA to treat the motor symptoms of PD. Pramipexole, ropinirole and piribedil are associated with side effects such as impulse control disorders and daytime drowsiness.^2,198^ Selective inhibition of Monoamine Oxidase B (MAO-B) increases the concentration of dopamine in the synaptic cleft.^199,200^

Amantadine, a weak noncompetitive NMDAR antagonist, effectively reduces LID by suppressing glutamatergic hyperactivity.^201^ Istradefylline, a Food and Drug Administration (FDA)-approved adenosine A2AR antagonist, is used as an add-on therapy to L-DOPA/carbidopa to reduce “off” episodes in PD, but is not effective as monotherapy.^197,200^ Although it is effective in combination therapy, its use is limited due to side effects such as exacerbation of dyskinesia and hallucinations.^202^

Clonazepam, a benzodiazepine derivative that acts as a GABA-A receptor agonist, is used clinically off-label for the treatment of RBD in PD by enhancing the inhibitory neurotransmission. Its use carries significant risks in terms of tolerance, dependence, and side effects, including daytime sedation and cognitive impairment.^203^ Atomoxetine shows promise in executive dysfunction,^204^ while droxidopa is effective for neurogenic orthostatic hypotension.^201^ Pimavanserin, a 5-HT2A inverse agonist, is FDA-approved for the treatment of PD psychosis and improves hallucinations without worsening motor function.^205^ Clozapine is effective for both psychosis and dyskinesia, but requires blood monitoring due to the risk of agranulocytosis.^202^ Muscarinic antagonists (e.g., trihexyphenidyl) alleviate tremor in younger patients, but are limited by cognitive side effects.^200^ Rivastigmine improves cognitive symptoms in PD’s dementia.^206^ Although current PD treatments focus on dopamine replacement, they do not have a disease-modifying effect and become ineffective over time, emphasizing the need for targeted approaches to improve symptom control and potential neuroprotection.

## 13 Conclusion

Parkinson’s disease is not solely associated with dopamine deficiency; it also involves dysfunction and imbalance in other neurotransmitter systems, including glutamate, GABA, serotonin, noradrenaline, and histamine. Examining these neurotransmitter imbalances at the molecular, cellular, and behavioral levels provides a more comprehensive understanding of Parkinson’s disease pathophysiology. Dysfunction in the serotonergic and histaminergic systems plays a critical role in the development of nonmotor symptoms, particularly sleep disturbances and L-DOPA-induced dyskinesia. A broader exploration of neurotransmitter involvement contributes to identifying novel therapeutic targets by uncovering the complex and interconnected mechanisms underlying Parkinson’s disease. PD is a multifactorial neurodegenerative disorder with complex pathophysiological changes that extend beyond dopaminergic dysfunction. While the loss of dopaminergic neurons in the substantia nigra remains the hallmark of the disease, growing evidence highlights the significant involvement of other neurotransmitter systems, including noradrenergic, serotonergic, cholinergic, glutamatergic, and GABAergic pathways, in disease progression and symptom variability. These nondopaminergic systems contribute to both motor and nonmotor symptoms including cognitive decline, mood disorders, and autonomic dysfunction, which remain inadequately addressed by current dopaminergic therapies. The interaction among neurotransmitter imbalances, α-syn pathology, mitochondrial impairment, oxidative stress, and neuroinflammation further complicates the disease outlook. This neurochemical complexity underlines the limitations of current treatments and the urgent need for disease-modifying strategies that target multiple neurotransmitter systems. Advances in neuroimaging and biomarker development hold promise for earlier and more precise diagnosis as well as for monitoring therapeutic efficacy. A comprehensive understanding of neurotransmitter dysregulation in PD may pave the way for more effective, individualized, and multifaceted therapeutic approaches. Future research should focus on elucidating these complex neurochemical interactions to guide the development of interventions capable of modifying disease trajectory. Finally, addressing the full spectrum of neurotransmitter dysfunction offers a path toward improved outcomes and the quality of life for individuals living with PD.

## Data Availability

The authors have nothing to report.

## Abbreviations

5-HIAA: 5-hydroxyindoleacetic acid
5-HT: Serotonin
5-HTP: 5-hydroxytryptophan
6-OHDA: 6-hydroxydopamine
7-NI: 7-nitroindazole
A-DA: anaerobic dopamine
A_1_R: A_1_ receptor
A_2_AR: A_2_A receptor
A_2_BR: A_2_B receptor
A_3_R: A_3_ receptor
ACh: acetylcholine
AD: Alzheimer’s disease
AMPAR: α-amino-3-hydroxy-5-methyl-4-isoxazolepropionic acid receptor
ASMT: acetylserotonin-O-methyltransferase
Aβ: amyloid β
BAC: bacterial artificial chromosome
BBB: blood–brain barrier
BDNF: brain-derived neurotrophic factor
BTLS: brain-targeted liposomal system
Ca^2+^: calcium
cAMP: cyclic adenosine monophosphate
CGINs: cholinergic and GABAergic striatal interneurons
cGMP: cyclic guanosine monophosphate
ChAT: choline acetyltransferase
Cl^–^: chloride ion
CNS: central nervous system
COMT: catechol-O-methyltransferase
D_1_R: D_1_ receptor
D_2_R: D_2_ receptor
D_3_R: D_3_ receptor
D_4_R: D_4_ receptor
D_5_R: D_5_ receptor
DA: dopamine
DBS: deep brain stimulation
DDC: dopa decarboxylase
DOPAC: 3,4-dihydroxyphenylacetic acid
DOPAL: 3,4-dihydroxyphenylacetaldehyde
EAAT: excitatory amino acid transporter
eNOS: endothelial nitric oxide synthase
EOPD: early onset Parkinson’s disease
EPN: entopeduncular nucleus
FDA: Food and Drug Administration
G6PD: glucose-6-phosphate dehydrogenase
GABA: γ-aminobutyric acid
GABA+: GABA and coregulated signals
GAD: glutamic acid decarboxylase
GAT-1: GABA transporter-1
GAT-3: GABA transporter-3
GAT: GABA transporter
Gi: inhibitory G protein
GLT-1: glutamate transporter-1
Go: other G protein
Gol, NO: Golgi-targeted fluorescent probe
GRM3: the mGluR3 receptor encoding gene
Gs: stimulatory G protein
GSH: glutathione
H-MRS: ultrahigh-field 14.1 T ^1^H magnetic resonance spectroscopy
H1R: histamine receptor 1
H2R: histamine receptor 2
H3R: histamine receptor 3
H4R: histamine receptor 4
HC: healthy controls
HCN: hyperpolarization-activated cyclic nucleotide-gated channels
Hcy: homocysteine
HDC: histidine decarboxylase
HMT: histamine *N*-methyltransferase
HPLC: high-performance liquid chromatography
IL-6: interleukin-6
iGluR: ionotropic glutamate receptor
iNOS: inducible nitric oxide synthase
K+: potassium
KAR: kainate receptor
L-DOPA: L-3,4-dihydroxyphenylalanine
LB: Lewy body
LC: locus coeruleus
LID: L-DOPA-induced dyskinesia
LN: Lewy neurites
LPS: lipopolysaccharide
M1R: muscarinic acetylcholine receptor 1
M2R: muscarinic acetylcholine receptor 2
M3R: muscarinic acetylcholine receptor 3
M4R: muscarinic acetylcholine receptor 4
M5R: muscarinic acetylcholine receptor 5
mAChR: muscarinic acetylcholine receptor
MAO-B: monoamine oxidase B
mDA: midbrain dopaminergic
mGluR: metabotropic glutamate receptor
mGluR3: metabotropic glutamate receptor 3
mPFC: medial prefrontal cortex
MPTP: 1-methyl-4-phenyl-1,2,3,6-tetrahydropyridine
MS-DB: medial septum-diagonal band
MSN: medium spiny neurons
MT1: melatonin receptor 1
MT2: melatonin receptor 2
mTOR: mechanistic target of rapamycin
Na^+^: sodium
NAA: N-acetyl-aspartate
nAChR: nicotinic acetylcholine receptor
NADPH: nicotinamide adenine dinucleotide phosphate
NE: norepinephrine
NET: norepinephrine transporters
NF-κB: nuclear factor-κB
NMDAR: *N*-methyl-d-aspartate receptor
nNOS: neuronal nitric oxide synthase
NO: nitric oxide
NOS: nitric oxide synthase
NOS1: nitric oxide synthase 1
NOS2: nitric oxide synthase 2
PD: Parkinson’s disease
PDT-: tremor absent
PDT+: tremor-dominant
PET: positron emission tomography
PIGD: postural instability gait difficulty
PKA: protein kinase A
PNS: peripheral nervous system
RBD: rapid eye movement sleep behavior disorder
RNS: reactive nitrogen species
ROS: reactive oxygen species
SERT: serotonin transporter
SNP: single nucleotide polymorphism
SNpc: substantia nigra pars compacta
SSD: somatic symptom disorder
STN: subthalamic nucleus
SWEDD: subjects with possible PD but no evidence of dopaminergic deficiency
TLR2: toll like receptor 2
TLR4: toll like receptor 4
TMN: tuberomammillary nucleus
TNF-α: tumor necrosis factor-α
TPH: tryptophan hydroxylase
TPH1: tryptophan hydroxylase 1
TPH2: tryptophan hydroxylase 2
TSE MRI: turbo spin–echo magnetic resonance imaging
VA: ventral anterior thalamic nucleus
VAChT: vesicular ACh transporter
VMAT2: vesicular monoamine transporter 2
VTA: ventral tegmental area
α-syn: α-synuclein
